# Identification of putative regulatory upstream ORFs in the yeast genome using heuristics and evolutionary conservation

**DOI:** 10.1186/1471-2105-8-295

**Published:** 2007-08-08

**Authors:** Marija Cvijović, Daniel Dalevi, Elizabeth Bilsland, Graham JL Kemp, Per Sunnerhagen

**Affiliations:** 1 Department of Cell and Molecular Biology, Lundberg Laboratory, Göteborg University, PO Box 462 SE-405 30 Göteborg, Sweden; 2 Department of Computer Science and Engineering, Chalmers University of Technology, SE-412 96 Göteborg, Sweden; 3 Max-Planck Institute for Molecular Genetics, Ihnestraße 63, D-14195 Berlin, Germany; 4 Biochemistry Department, University of Cambridge, 80 Tennis Court Road, Cambridge CB2 1GA, UK

## Abstract

**Background:**

The translational efficiency of an mRNA can be modulated by upstream open reading frames (uORFs) present in certain genes. A uORF can attenuate translation of the main ORF by interfering with translational reinitiation at the main start codon. uORFs also occur by chance in the genome, in which case they do not have a regulatory role. Since the sequence determinants for functional uORFs are not understood, it is difficult to discriminate functional from spurious uORFs by sequence analysis.

**Results:**

We have used comparative genomics to identify novel uORFs in yeast with a high likelihood of having a translational regulatory role. We examined uORFs, previously shown to play a role in regulation of translation in *Saccharomyces cerevisiae*, for evolutionary conservation within seven *Saccharomyces *species. Inspection of the set of conserved uORFs yielded the following three characteristics useful for discrimination of functional from spurious uORFs: a length between 4 and 6 codons, a distance from the start of the main ORF between 50 and 150 nucleotides, and finally a lack of overlap with, and clear separation from, neighbouring uORFs. These derived rules are inherently associated with uORFs with properties similar to the *GCN4 *locus, and may not detect most uORFs of other types. uORFs with high scores based on these rules showed a much higher evolutionary conservation than randomly selected uORFs. In a genome-wide scan in *S. cerevisiae*, we found 34 conserved uORFs from 32 genes that we predict to be functional; subsequent analysis showed the majority of these to be located within transcripts. A total of 252 genes were found containing conserved uORFs with properties indicative of a functional role; all but 7 are novel. Functional content analysis of this set identified an overrepresentation of genes involved in transcriptional control and development.

**Conclusion:**

Evolutionary conservation of uORFs in yeasts can be traced up to 100 million years of separation. The conserved uORFs have certain characteristics with respect to length, distance from each other and from the main start codon, and folding energy of the sequence. These newly found characteristics can be used to facilitate detection of other conserved uORFs.

## Background

The expression of protein-coding genes in eukaryotes is regulated on several levels even after the transcript has been formed. Translation into protein requires assembly of ribosomes with initiation factors on the mRNA in the 5'-untranslated region (5'-UTR) near the initiation codon. After completion of a translation round, at the stop codon, termination factors cause the ribosome to dissociate and fall off the template. Scanning of the mRNA by the ribosome from its 5' end is seen as the major mechanism for locating the start codon of the main ORF [1]. In several cases, one or several ORFs are present in the 5'-UTR. Such uORFs can negatively regulate translation of the main ORF by interfering with reassembly of the initiation complex at its start codon. Conceptually, this could occur through several mechanisms (for review, see [2,3]). The ribosome could remain bound to the mRNA downstream of the uORF, blocking further rounds of translation. In at least one case in yeast, *CPA1*, it has been convincingly shown that missense mutations at internal positions in the uORF abolish its function, implying that the uORF-encoded peptide is important for the effect on translation [4]. The working model proposes that the newly synthesised peptide blocks progression of the ribosome. There is recent evidence that such stalling induces the nonsense-mediated mRNA decay (NMD) pathway [5]. Yeast *GCN4 *is the best-investigated case of translational control through uORFs; in this case however, the encoded peptide is not invoked to play a functional role [6]. *GCN4 *translation is controlled by four uORFs. Reinitiation downstream of uORF1 occurs at different distances from its stop codon depending on the cellular levels of eIF2-GTP bound to Met-tRNA (ternary complex). If this level is high, reinitiation will most frequently occur upstream of uORF4. The sequence downstream of uORF4 is unfavourable for reinitiation, and so translation of the main ORF is prevented. With low levels of ternary complex, uORF4 will be bypassed and the main ORF translated [7]. For other genes, a negative correlation between the length of the uORF and frequency of downstream initiation has been observed [8].

Comparative genomics has emerged as a main instrument to discern important structural and regulatory elements in nucleic acid sequences. The optimal evolutionary distance between genomes to be compared depends on the property under investigation. Functional protein domains can be conserved throughout the eukaryotic kingdom and beyond, whereas regulatory *cis-*elements in DNA diverge much more rapidly, and thus require comparisons between closely related species for efficient detection. Genomes from the *Saccharomyces sensu stricto *group and more distantly related *Saccharomyces *species have been successfully employed to identify transcription factor binding sites in promoters [9-12]. Among these species, *S. paradoxus*, *S. mikatae*, *S. bayanus*, and *S. kudriavzevii *(all members of the *Saccharomyces sensu stricto *group) diverged from *S. cerevisiae *between 5 and 20 million years ago, while *S. castellii *and *S. kluyveri *are considerably more distant, with an estimated divergence around 100 million years ago [13]. Beside conservation of sequence, conservation of position and order (synteny) of genes or sequence elements can be used as a powerful complementary approach to identification in a complex genomic context, as has been shown for gene finding in the rat genome using alignments with the human and mouse counterparts [14]. Comparative genomics of three closely related species of *Aspergillus *has been attempted to predict functional uORFs [15], and the same approach was used comparing human and mouse genomic sequences [16]. Another analysis was recently performed using a comparison of seven *Saccharomyces *species' genomes to identify tentatively functional uORFs [17].

The present investigation combines two independent criteria for assessing the potential for a uORF to be functional in regulation: evolutionary conservation of sequence and position on one hand; and conformity to certain properties, that we have found to be associated with characterised uORFs with a regulatory role, on the other. The latter have been coded into a scoring system, which we have used to rank uORF candidates in the *S. cerevisiae *genome. We have found 379 uORFs in 252 genes that fulfil these criteria, and which we predict to be functional. Of these, 16 genes have previously been characterised at the translational level, and 7 of these contain 12 uORFs with regulatory roles. The remaining 367 uORFs identified in this study are novel. Since ranking according to our scoring system identifies novel uORFs with a better than average evolutionary conservation, we infer that this combined approach is efficient.

## Results

### Conservation of uORFs in GCN4 homologues in other fungi

**Figure 1:**
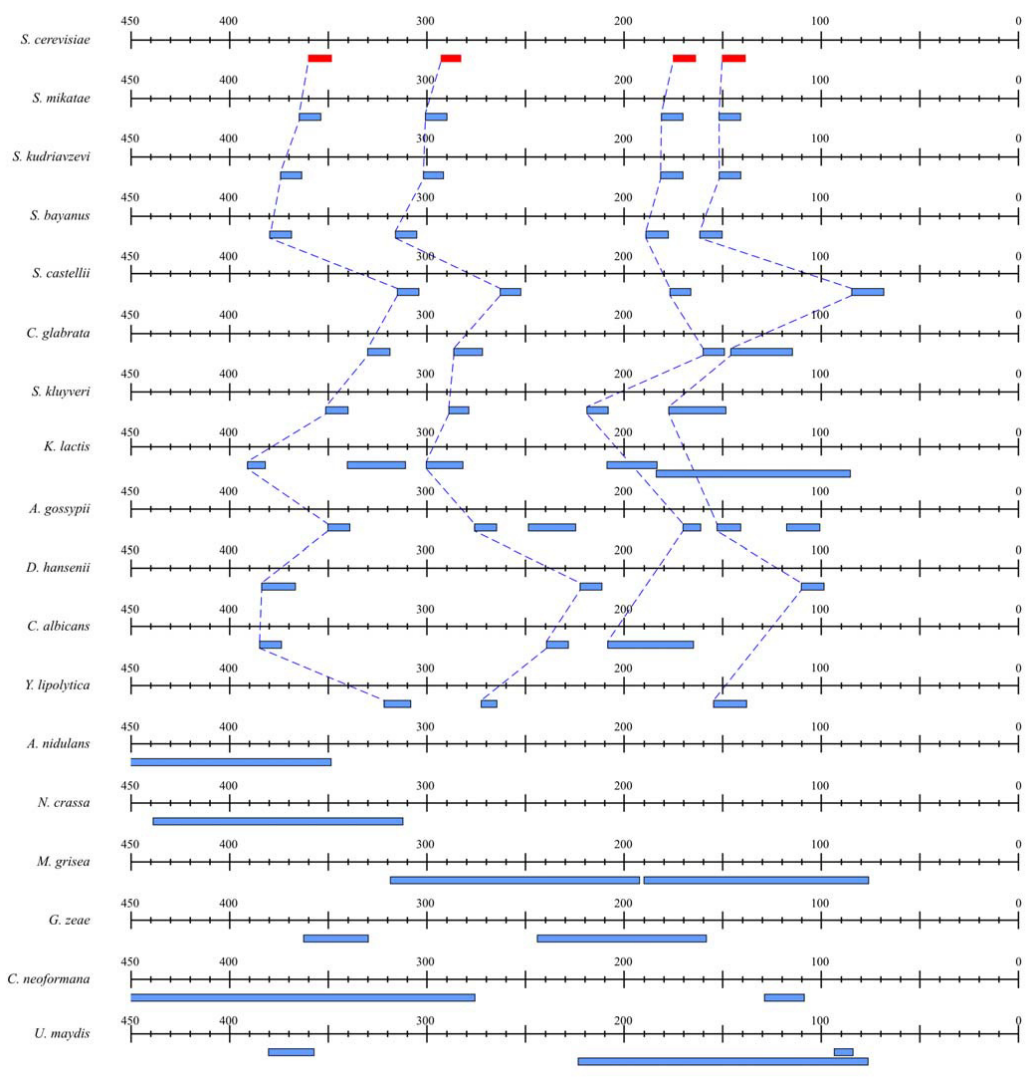
Conservation of uORFs in the *GCN4 *locus of *S. cerevisiae *and homologues in 18 fungal species. The species are ordered approximately according to evolutionary distance from *S. cerevisiae *[13]. uORFs which are conserved with respect to sequence and position within the 5' flanking region are connected by dotted lines. The start codon of the *GCN4 *coding sequence is located at position 0.

To estimate the degree of evolutionary conservation of functional uORFs among fungal species, we decided to initially investigate the homologues of the *GCN4 *locus, which is well-characterised in *S. cerevisiae *with respect to the regulatory role of its four uORFs [6]. Using WU-BLAST2-TBLASTN at SGD, we identified *GCN4 *orthologue candidates in 18 fungal species. In all cases it was possible to find one unambiguous homologous locus. All upstream regions were aligned, and uORFs were examined for similarity in sequence and distance from the main ORF (Fig. 1). All four uORFs are well conserved in all species up to and including *Ashbya gossypii*, with the sole exception of *Kluyveromyces lactis*. uORFs 1, 2, and 4 have discernible homologues at even longer evolutionary distances, as far as *Yarrowia lipolytica *(representing a split of > 200 MYr [13]). In even more distantly related fungi, representing basidiomycetes and filamentous ascomycetes, no homologous uORFs were found, however. These findings demonstrate that uORFs with a proven regulatory role in *S. cerevisiae *are indeed conserved in genomes throughout most of *Hemiascomycetes*. It is thus a reasonable expectation to find conservation of uORFs with a regulatory role among *Saccharomyces *sister species, and to use this as a criterion for classifying them as functional.

### Conservation between species among previously recognised uORFs

**Figure 2:**
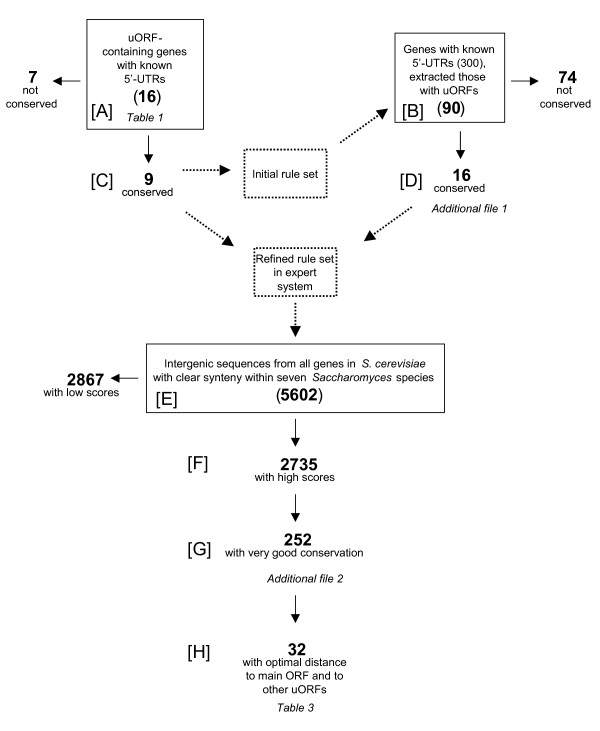
Flowchart of the steps in defining criteria to find novel uORFs that share characteristics with known functional uORFs. Solid arrows denote partition of a gene set into subsets; dotted arrows denote that a gene set or an algorithm is influenced by or operates on something. Letters within brackets identify the different subsets referred to in the text. Set A was the initial training set; set A + B was the training set for the refined rule set.

**Figure 3:**
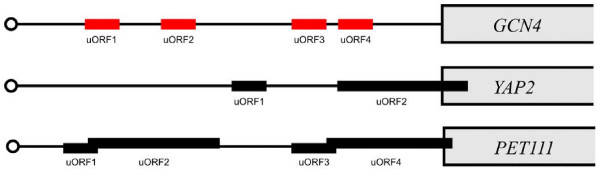
Three major classes of organisation of uORFs found in the *S. cerevisiae *genome. Not drawn to scale.

The starting point for our investigation was a set of 16 *S. cerevisiae *genes with characterised 5'-UTRs containing uORFs [3], (Fig. 2, set A). Investigation of this set revealed 27 uORFs, for an average of 1.8 uORFs per gene. A summary of the properties of this set is found in Table 1. Among this set of uORFs, we discerned three subclasses with respect to their length and positioning (Fig. 3). The first and most abundant subclass, typified by *GCN4*, has short uORFs that do not overlap either with each other or with the main ORF. The second class, which includes *YAP2*, has short as well as longer uORFs, which overlap with the main ORF but not with each other. The third class, represented here by *PET111*, has short and long uORFs that overlap both with each other and with the main ORF.

**Table 1:** Evolutionary conservation of uORFs highlighted by Vilela and McCarthy [3]. Genes with conserved uORFs are shown in bold.

Gene	uORF conservation^1^	If predicted not to be functional, reason for this	Evidence about functional role
***CLN3***	yes (1/1; 4/6)		[26]
***GCN4***	yes (4/4; 7/7)		[6]
*INO2*	no (0/1; 0/6)	uORF too long	
*PPR1*	no (0/1; 0/6)	uORF too close to main AUG	
*SCO1*	no (0/1; 0/5)	uORF too close to main AUG	[32]^2^
***CPA1***	yes (1/1; 5/5)		[4]
***HAP4***	yes (2/2; 4/4)		[43]^3^
*LEU4*	no (0/1; 0/7)	uORF too close to main AUG	
***TIF4631***	yes (4/6; 4/6)		[31]^3^
***YAP1***	yes (1/1; 3/5)		[27]
***YAP2***	yes (2/2; 3/3)		[27]
*CBS1*	no (0/1; 0/5)	uORF too close to main AUG	[32]^2^
*DCD1*	no (0/1; 0/7)	uORF too close to main AUG	
***HOL1***	yes (1/1; 4/4)		[29]
***PET111***	yes (3/4; 3/4)		[30]^4^
*SCH9*	no (0/1; 0/6)	uORF too long (55 codons)	

The *STA1-3 *genes mentioned by Vilela and McCarthy are not present in the standard S288c genome sequence and were not included in this analysis.

^1 ^Numbers between parentheses denote: (number of uORFs conserved/total number of uORFs; number of species where uORFs are conserved/total number of species where orthologue could be identified)

^2 ^Evidence **against **translational control by uORFs

^3 ^Evidence for translation using an IRES mechanism

^4 ^Pet111 controls translation of another mRNA, but no evidence for uORF control of *PET111 *expression

**Figure 4:**
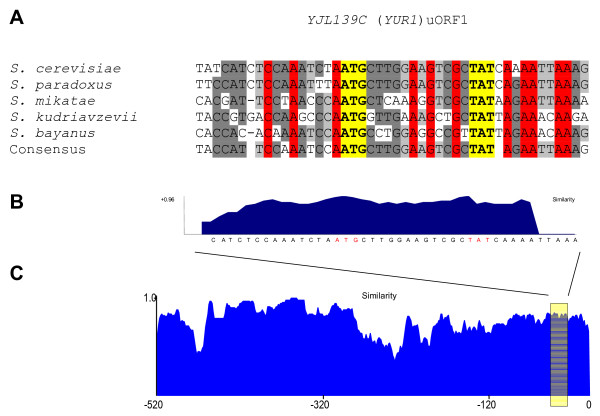
Alignment of a region containing uORF1 (closest to the start codon of the main ORF) from *S. cerevisiae YJL139c *(*YUR1*) with the orthologous sequences from four other *Saccharomyces *species. **A**, sequence alignment. The start and stop codons of the uORF are marked in yellow. **B**, DNA sequence similarity profile of uORF1. **C**, DNA sequence similarity profile of the entire 5'-UTR of *YUR1 *and its homologues.

To investigate to which extent these uORFs are conserved, we aligned the sequences from 1000 bp upstream of the start codon of each of these *S. cerevisiae *genes with their orthologues from the other members of the *Saccharomyces sensu stricto *group, plus *S. castellii *and *S. kluyveri *(for an example of visualisation of an alignment, see Fig. 4). The result is shown in Table 1. Nine of the 16 genes (*CLN3*, *CPA1*, *GCN4*, *HAP4*, *HOL1*, *PET111*, *TIF4631*, *YAP1*, *YAP2*) turned out to possess uORFs that are visibly conserved in most other *Saccharomyces *species where an orthologue could be identified. As expected, there was generally a gradual decline of conservation with increasing evolutionary distance. Thus, all 18 uORFs were conserved in *S. paradoxus*, *S. mikatae*, and *S. bayanus*; 10 were conserved in *S. castellii*, 8 in *S. kudriavzevii*, and 3 in *S. kluyveri*.

An analysis of common properties of the 9 genes, where conservation of uORFs was evident, showed two features that the majority of them share, and which might be used to distinguish them from spurious uORFs. First, the uORFs are short, on average 6.5 codons, to be compared with the average of 12.9 codons for all uORFs in this set, and 15.0 codons for the non-conserved uORFs. Second, the most downstream uORF is placed not closer than 50 nt from the start codon of the main ORF; in most cases at a distance between 50 and 150 nt.

### Extension of heuristics for classification of functional uORFs in a larger dataset

In the second step, we extended our analysis to the whole collection of *S. cerevisiae *genes for which the extent of the 5'-UTR is known [18]. All 294 5'-UTR sequences were downloaded from the UTRResource database and analysed for their uORF content. In 90 of these genes, at least one uORF was found (Fig. 2, set B). The corresponding sequences from the other genomes were aligned as previously. Out of these 90 genes, 16 were found to contain at least one conserved uORF (average 1.7 uORF per gene; Fig. 2, set D). The properties of uORFs, both conserved and non-conserved, in this set are summarised in Table 2, and the 16 genes with conserved uORFs detected in this work are listed in additional file 1.

**Table 2:** Properties of uORFs found in 294 previously identified 5'-UTRs [18], after classification as evolutionarily conserved or non-conserved.

	Conserved	Non-conserved
Total number	**16**	**74**
Average length (codons)	**5.1**	**15.4**
Average distance from start codon of main ORF	**61**	**121**

We then reanalysed the combined set of 106 (16 + 90; Fig. 2, set A + B) uORF-containing 5'-UTRs, again looking for features that distinguish uORFs of the 25 (9 + 16; set C + D) 5'-UTRs where evolutionary conservation was detected, from those without detectable conservation.

### Creation of an expert system and its implementation to discriminate functional from spurious uORFs on a genome-wide level

**Figure 5:**
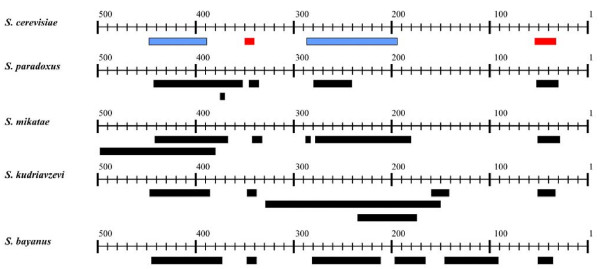
Schematic of the arrangement of uORFs in the 5' flank of *S. cerevisiae YJL139c *(*YUR1*) and its homologues in other *Saccharomyces *species. This type of diagram is produced automatically for each gene, showing the intergenic sequence as a numbered axis; the coding sequence of the gene starts at the position one of the intergenic sequence. uORFs are shown as boxes. The box colours show *S. cerevisiae *uORFs predicted to be functional (red), or not functional (blue). uORFs from other species are represented by black boxes, since we do not predict their functionality. The rightmost uORF (uORF1) is identical to the one shown in Fig. 4.

We wanted to perform an analysis of all 5'-flanking sequences of recognised genes in the *S. cerevisiae *genome, using the approximate criteria that we derived from the set of conserved uORFs in characterised 5'-UTRs. For this, we needed a formal implementation of criteria, which was also able to perform a genome-wide scan in a reasonable time. We used an expert system (see Materials and Methods) where the following rules, derived from the analysis of the 106 genes with conserved uORFs (Fig. 2, set A + B), were encoded. The system gave as an output a numeric score for each uORF based on: a) the length of the uORF (optimal 4 – 6 codons); b) the distance of the gene-proximal uORF (optimal 50 – 250 nt); c) the number of uORFs upstream of a main ORF (optimal < 10). These values were stored in frames structures in an expert system shell. A score (cf) for each uORF was deduced using a set of production rules with associated cfs, and the highest score among the uORFs upstream of a certain gene was assigned to that gene. A diagram visualising the length, position, certainty factor and conservation in other *Saccharomyces *species is produced automatically for each gene (Fig. 5). We analysed a total of 5602 intergenic sequences of recognised genes from *S. cerevisiae *(Fig. 2, set E). As in most cases the length of the 5'-UTR was unknown, the entire intergenic sequences were used. Among these sequences, a total of 51904 potential uORFs were found. In our scoring system, 24449 uORFs distributed among the 5' flanks of 2735 genes (set F) were assigned a cf ≥ 0.98.

### Conservation of uORFs that conform to newly derived rule set

We extracted the intergenic region from each of the 2735 genes and aligned them to their counterparts from the other 6 *Saccharomyces *species as described above. uORFs from *S. cerevisiae *with scores above 0.7 were visualised by colour-coding (red, see Fig. 2). We manually examined all alignments. We found 379 uORFs distributed among 252 genes (Fig. 2, set G) to show a clear conservation of sequence and position in at least 4 species. The mean score of these genes was 0.98, notably higher than the average score of the entire set (-0.09), and the average score of the genes selected for inspection of alignment (-0.005).

The fact that uORFs with a high score were significantly better conserved indicates that the rules of our scoring system are indeed detecting features that have been conserved in evolution, and by inference, are likely to play a functional role. Out of the 16 previously characterised genes with uORFs (Table 1), 9 are conserved as previously mentioned, and 7 out of these 9 (*CLN3*, *GCN4*, *HAP4*, *HOL1*, *PET111*, *YAP1*, *YAP2*) were also found in the list of 252 genes with uORFs that we identified in the screen described above. By contrast, for a group of 40 randomly selected genes (with an average score of -0.09), the degree of conservation of uORFs was 11.4% in *S. paradoxus*; 2.4% in *S. mikatae*, 5.2% in *S. bayanus*, 2.3% in *S. castellii*, 6.7% in *S. kudriavzevii*, and 5.2% in *S. kluyveri*. The fact that the degree of conservation does not follow the evolutionary closeness between species is a sign that this does not reflect actual conservation of sequences. It should be noted that for *PET111 *and *YAP2*, only the shorter uORFs that do not overlap with the main ORF (*PET111 *uORF1 and uORF3; *YAP2 *uORF1; Fig. 3) received high scores. The complete list comprising 252 genes with conserved uORFs predicted to be functional is shown in additional file 2.

In the course of our work, the study by Zhang and Dietrich [19] verified the 5' ends of a large set of *S. cerevisiae *mRNAs, 24 of which were shown to contain uORFs (additional file 3). We did not use these to modify our rule set, but examined to what extent they are conserved and predicted to be functional according to our work. The uORFs of three genes (*AGE1*, *PIC2 *and *PCL5*) are conserved and conform well to our rule set; those of another two (*AMN1 *and *URA5*) are conserved but get lower scores since they deviate too much from the optimal length. Out of the remaining 19 genes, the uORFs are not conserved in other species (17 genes), no orthologues were found (*IMD1*), or no uORF was found at the indicated position (*YNR034W-A*).

### Sequence properties of conserved uORFs

Having identified a large set (379) of uORFs predicted to have biological function, we analysed these for common properties. First, we noted that there is no correspondence between the reading frames of functional uORFs and the frames of either the main ORF or of other uORFs upstream of the same gene.

We noticed that a marked feature of uORFs with a high score and a high degree of conservation was a clear physical separation from other, low-scoring (and by inference spurious), uORFs. In our set of 252 genes, the average distance between a predicted functional uORF and another neighbouring functional uORF is 127 nt, whereas the average distance between a uORF predicted to be functional and its closest neighbouring non-functional uORF is 100 nt. A genome-wide investigation of all intergenic regions in *S. cerevisiae *of the average distance between neighbouring uORFs gave the value 79 nt. This indicates that functional uORFs are indeed characterised by having a wider uORF-free zone around them than spurious uORFs. Therefore, we decided to add this criterion to augment the process of ranking uORFs according to the likelihood of them having a functional role. From the group of 252 genes with high scores, we manually selected 32 cases (Fig. 2, set H) with the following properties: a) the uORF responsible for the high score given to that 5' flanking sequence was well separated from other uORFs with low scores, b) optimal distance from main ORF, c) optimal length. This conforms to the properties of the 9 + 16 (set C + D) conserved uORFs that we initially identified. Of these 34 uORFs from 32 genes, all 34 (100%) are conserved in *S. paradoxus*, 29 (83%) in *S. bayanus*, 23 (66%) in *S. kudriavzevii*, 14 (40%) in *S. castellii*, and 3 (9%) in *S. kluyveri*. In the *S. mikatae *genome sequence, syntenic homologues could be identified for only 16 out of the 32 genes, and all 16 of these (100%) had conserved uORFs. These 32 genes, shown in Table 3, represent the cases where we make the strongest prediction for the presence of functional uORFs with a regulatory role. The uORFs in this sub-group are better conserved than the average in the group comprising 252 genes that they were selected from. In this larger set, only 85% of uORFs were conserved in *S. paradoxus*, 43% in *S. mikatae*, 37% in *S. bayanus*, 37% in *S. kudriavzevii*, 20% in *S. castellii*, and 11% in *S. kluyveri*.

**Table 3:** 32 newly identified genes with highly conserved uORFs strongly predicted by the rule set to be functional (marked in bold), with an optimal spacing to the main ORF and other uORFs. Numbering of uORFs is 3' to 5', as uORFs were found from intergenic sequences.

**ORF**	**Name**	**Length (nt) of intergenic sequence**	**Number and size (codons) of uORFs**	**Position (nt) relative to start codon of main ORF**	**Predicted length (nt) of 5'-UTR [20]^a^**
***YDL146W***	***LDB17***	489	**uORF1(3**)	-53	**514**
***YDL176W***	**Uncharacterised**	376	**uORF1(9)**	-104	**157**
***YDL205C***	***HEM3***	860	**uORF1(8)**	-130	**183**
***YEL061C***	***CIN8***	509	**uORF1(4)**	-109	**128**
***YER167W***	***BCK2***	757	**uORF1(7)**	-245	**286**
***YGL006W***	***PMC1***	387	**uORF1(4)**	-144	**438**
***YJL139C***	***YUR1***	505	**uORF1(8)**	-55	**60**
***YKL182W***	***FAS1***	1030	**uORF1(6)**	-142	**548**
***YLR047C***	***FRE8***	825	**uORF1(3)**	-230	**264**
***YLR427W***	***MAG2***	315	**uORF1(6)**	-71	**109**
***YMR145C***	***NDE1***	1006	**uORF1(4)**	-165	**215**
***YNL053W***	***MSG5***	358	**uORF1(4)**	-104	**120**
***YNL094W***	***APP1***	771	**uORF1(3)**	-155	**278**
***YNR016C***	***ACC1***	1539	**uORF1(3)**	-342	**540**
***YOL100W***	***PKH2***	1317	**uORF2(9)** **uORF1(7)**	-338 -81	**255**
***YOL130W***	***ALR1***	1042	**uORF1(5)**	-103	**290**
***YOR061W***	***CKA2***	371	**uORF1(5)**	-103	**162**
***YOR124C***	***UBP2***	388	**uORF1(7)**	-211	**250**
***YOR137C***	***SIA1***	628	**uORF1(9)**	-367	**440**
**YOR231W**	***MKK1***	488	**uORF1(9)**	-72	**148**
**YOR254C**	***SEC63***	248	**uORF1(5)**	-82	**100**
**YPL057C**	***SUR1***	373	**uORF1(5)**	-145	**253**
**YPR026W**	***ATH1***	821	**uORF1(4)**	-65	**112**
***YER118C***	***SHO1***	441	**uORF1(8)**	-212	206
*YEL013W*	*VAC8*	541	**uORF2(3)** **uORF1(3)**	-364 -306	252
*YEL026W*	*SNU13*	692	**uORF3(3)** uORF2(2) uORF1(2)	-206 -198 -193	170
*YLR009W*	*RLP24*	454	**uORF1(3)**	-220	171
*YLR243W*	Uncharacterised	320	**uORF1(3)**	-111	80
*YML093W*	*UTP14*	241	**uORF1(4)**	-99	21
*YMR215W*	*GAS3*	313	**uORF1(4)**	-147	62
*YNL229C*	*URE2*	457	uORF2(11) **uORF1(5)**	-384 -285	217
*YNR049C*	*MSO1*	393	**uORF1(3)**	-142	123

^a ^Genes where predicted functional uORFs are located within the estimated 5'-UTR are marked in bold.

Since we used genomic DNA to derive the uORFs for this study, it is important to consider whether they lie within the transcribed region (5'-UTR) of the gene in question. We manually examined the position of the 34 top-scoring uORFs (set H) using data from the recently published high-density *S. cerevisiae *transcriptome map obtained from tiling arrays [20]. In 23 of the 34 cases, the genomic uORF was unambiguously placed within the transcribed region (the corresponding genes marked in bold in Table 3), and in one additional case (*SHO1*), it is quite close to the predicted transcript start site. To determine to what extent genomic uORFs not predicted to be functional were transcribed, we picked 40 uORFs with the lowest score, on average located at the same distance from the start codon of the main ORF (250 nt) as the 34 uORFs in the top group in Table 3. In stark contrast, only 20% of these low-scoring uORFs were located within transcripts.

The A/T-rich sequence downstream of *GCN4 *uORF1 and the G/C-rich sequence downstream of *GCN4 *uORF4 have been proposed to be essential for their translational regulatory properties. Therefore, we also compared the G/C content of the 20 nt immediately upstream and downstream of all uORFs in the whole genome with those from the top-scoring 32 genes where uORFs in addition have an optimal distance to the main ORF and a clear separation between uORFs (Fig. 2, Table 3). We found an average G/C content of 38.6% upstream of high-scoring uORFs (vs. 36.9% for all uORFs in the whole genome), and 36.9% downstream of uORFs (vs. 36.3% for all uORFs in the whole genome). We conclude that there is no significant deviation in G/C content from the genome average for sequences flanking functional uORFs.

Finally, we examined the sets of genes carrying candidate functional uORFs found in this work for the predicted folding energies of their 5'-UTRs. It has been shown that 5'-UTRs generally are more weakly folded than bulk or randomised sequences, and that strongly translated mRNAs tend to be even less folded [21]. We found that the predicted folding energies of the 200 nt immediately preceding the AUG of the main ORF were weaker for the initial set of genes containing previously recognised functional uORFs than for the average gene (Table 4). Interestingly, our newly found genes containing uORFs predicted here to be functional also have weaker folding energies in this region; most significantly for the 32 most highly ranked genes, and to a lesser extent also the larger set of 252 genes (Table 4).

**Table 4:** Calculated minimum free folding energy of the 200 nt immediately upstream of the start codon of different sets of uORF-containing genes [21].

Set	Minimum free energy (kcal/mol)
9 genes in initial set with conserved uORFs (Table 1; Fig. 2 set C)	-25.8
32 genes with highly conserved uORFs with optimal spacing (Table 3; Fig. 2 set H)	-32.8
252 genes with highly conserved uORFs (additional file 2; Fig. 2 set G)	-35.4
All genes in genome	-36.6

### Possible role of peptide product of predicted functional uORFs

We then wanted to estimate the prevalence among regulatory uORFs of mechanisms that depend on the encoded peptide. We reasoned that if the encoded peptide is relevant, this should be reflected by the absence of frameshift mutations (*e.g*. one +1 followed by a -1 frameshift, thus preserving the length of the uORF but altering the peptide sequence) and by a high ratio of synonymous to non-synonymous mutations (d_s_/d_n_), similar to other protein-coding sequences. Among the 34 uORFs we investigated (from the 32 genes in Table 3), we found one case of frame-shifts within one uORF, namely *YER118c *in *S. kudriavzevii*. As a complementary approach, we calculated the ratio of synonymous to non-synonymous substitutions in uORFs by comparing the orthologous sequences of *S. cerevisiae*, *S. paradoxus*, *S. mikatae*, and *S. bayanus*. For the uORFs in Table 3, the d_s_/d_n _ratio calculated from a total of substitutions is 0.41. This is significantly lower than the average d_s_/d_n _value we determined from 3268 protein-coding sequences from the same species, namely 1.80.

As a further estimation of the likelihood that uORFs encode a functional peptide, we compared the codon adaptation index (CAI; [22]) of the set of 252 conserved uORFs in additional file 2 (CAI = 0.151) with those of the entire group of 24449 uORFs (mostly non-functional; CAI = 0.149). This is to be contrasted with the indices for weakly (CAI = 0.19) and highly (CAI = 0.77) expressed protein-coding main ORFs [23]. There is thus no bias for a higher CAI in the conserved uORFs examined.

The sequences around the start codon that promote efficient translation are much less frequent in uORFs than in main ORFs [24]. In accordance with this, we did not find good fits to the consensus found for *S. cerevisiae*, (A/U)A(A/C)AA(A/C)AUGUC(U/C, [25]) in most high-scoring uORFs. For the positions with the greatest impact on translational efficiency, the base frequencies as calculated from the set of 252 genes were not significantly different from bulk DNA: at -3; 35% A, 16% C, 20% G, 29% T; at +4; 32% A, 22% C, 17% G, 29% T.

### Biological context of genes with predicted functional uORFs

In order to identify any common denominator for the biological function of these 252 genes, we performed a Gene Ontology (GO) term analysis at SGD. There was no single term unifying the majority of the genes; however there was a moderate overrepresentation of genes with the function "transcription regulator activity" (9.6% vs. 4.4% in the whole genome; P = 3.1 × 10^-4^); see Table 5. There was also an overrepresentation of the cellular process "development" (10.4% vs. 5.4%; P = 10^-3^). The genes associated with "development" are mainly involved in establishment of cell polarity and sporulation. Related to this, we also noted an overrepresentation of genes with a role in pseudohyphal growth (2.4% vs. 0.6%; P = 7 × 10^-3^), even though this category is not classified under "development" in GO. Most of the genes for pseudohyphal growth are also included in one of the other categories (cell polarity, transcription); see Table 5.

**Table 5:** Major functional classes for genes that harbour conserved uORFs predicted to play a regulatory role (Fig. 2, set G).

**Sporulation**	**Transcription**	**Filamentous growth**	**Cell polarity**
	*FKH2*		
	*FLO8*		
	*SOK2*		
BDF1			
		*BUD8*	
		*CDC42*	
*ADE16*	*CAT8*	*SHO1*	*BUD6*
*MDS3*	*ELP3*		*BUD22*
*MSO1*	*GCN4*		*CDC12*
*PRE1*	*HAP4*		*CKA2*
*RIM9*	*HFI1*		*HKR1*
*SMK1*	*MET32*		*RHO3*
*SSP1*	*RRN10*		
	*RRN11*		
	*RCS1*		
	*SIF2*		
	*SKN7*		
	*SOK2*		
	*SPT8*		
	*SRB7*		
	*SUT1*		
	*SWI5*		
	*TAF3*		
	*TAF12*		
	*URE2*		

## Discussion

### Properties of conserved uORFs

The independent properties that correlate with the newly found evolutionarily conserved uORFs are: a) short length, 4–6 codons; b) distance from main ORF between 50 and 250 nt; c) a distance to the nearest conserved uORF slightly greater than between neighbouring spurious uORFs; d) weaker folding energies of the most downstream 200 nt of the 5'-UTR than for the average gene; e) a 3-fold higher probability of being located within a transcript than randomly chosen uORFs in the genome at an equivalent distance from the main ORF. The first two of these features emerged from our evolutionary comparison of the initial set of uORFs with experimentally demonstrated regulatory function, where it was shown that conserved uORFs had these properties. These two rules were then used to rank all uORFs in the genome, facilitating the manual inspection of alignments with homologous regions from other genomes to reveal evolutionary conservation. The last three properties of evolutionarily conserved uORFs became apparent in the final analysis of the larger set of novel predicted functional uORFs. We believe that these rules of thumb can be helpful in the identification of functional uORFs from other genomes.

Several factors underpin the approach we have used for discrimination of uORFs with a regulatory role from those arising in the genome by chance. The set of genome sequences from seven *Saccharomyces *species utilised in this work lends itself well to extracting putative *cis*-regulatory elements with bioinformatics methods. The reasons for this are threefold: a) the species represent a range of rather short evolutionary distances, suitable for detection of sequence features that change relatively rapidly; b) budding yeast genomes are less complex than those of most eukaryotes, with *e.g*. fewer repetitive elements and protein binding sites, and have short intergenic sequences; c) using seven genomes for comparison is inherently more powerful than two, such as man vs. mouse [16] or three *Aspergillus *species [15]. Independently of the criterion of evolutionary conservation, we have developed a set of heuristic rules of length and spacing of uORFs, which we have used to pre-sort the 51904 uORFs found in the *S. cerevisiae *genome, in order to be able to concentrate efforts on the best candidates. Lastly, the visualisation tool we constructed allows immediate spotting of conserved uORFs in other species among candidate uORFs.

It is noteworthy that among the 9 genes in the initial set where conservation of uORFs was found, there is evidence in the literature for a regulatory role of uORFs in six cases: *GCN4 *[6], *CLN3 *[26], *YAP1 *[27], *YAP2 *[27,28], *HOL1 *[29], and *CPA1 *[4]. We note that the uORFs of five out of these six genes (all except *CPA1*) were identified as functional by our automated scoring system. *CPA1 *was not identified is because its uORF is much longer (20 nt) than the optimum in our scoring system (4 – 6 nt). The *CPA1 *uORF also belongs to a different functional class, where the encoded peptide has a direct role in the regulatory mechanism [4], in contrast to the *GCN4*-like uORFs that likely make up the vast majority in the set we identified. Of the remaining three genes, *PET111 *is an interesting case in that it has been recognised that Pet111p acts to control translation of another mRNA, namely the mitochondrially encoded *COX2 *[30]. To our understanding, post-transcriptional control of *PET111 *itself by uORFs has not been considered, however. For *TIF4631*, itself encoding a translation factor, translational control through an internal ribosome entry site (IRES) mechanism has been argued [31], but we are not aware that uORF-mediated control has been demonstrated. For *HAP4*, finally, we have not been able to find documentation in the literature about regulation through uORFs. Considering the genes where no conserved uORFs were found, in fact there are reports in the literature indicating that the uORF is *not *functional for two of them: *CBS1 *and *SCO1 *[32].

It is relevant to compare the results of our investigation with those of Zhang and Dietrich [17]. There, a list of 15 genes containing 19 newly predicted functional uORFs is presented (additional file 4). Six of these (*FOL1*, *HEM3*, *MBR1*, *MKK1*, *RPC11*, *WSC3*) are also highly ranked (score ≥ 0.98) with our methods; one of them (*HEM3*) is in our top list (Table 3). Of the remaining eight, several observations may explain why they were not highly scored by our methods. One gene, *IMD4*, is not present in other fungal genomes, and is given a low score by our methods since the uORF is too long. For five genes (*AVT2*, *TPK1*, *APC2*, *SPE4*, *SPH1*) the distance to the main ORF is too short. Two further genes have three uORFs each, and not all of them are conserved. Thus, uORF2 of *ARV1 *is conserved and gets an intermediate score, because it is too long, whereas the other uORFs are not conserved; uORF2 and uORF3 of *SLM2 *are conserved and get high scores whereas uORF1 is not conserved. Zhang and Dietrich [17] used evolutionary conservation as the sole criterion for inclusion in the set to be considered. Because of the very large number of genes and uORFs to be investigated, we believe it is efficient to concentrate manual inspection of alignments to the cases with the highest likelihood of constituting true regulatory uORFs. We think this is the reason why we succeeded in identifying a much larger set of candidates in this work (252 vs. 35). We have noted that the average length of the *S. cerevisiae *5'-UTRs as measured by David et al. (260 nt; [20]), is higher as earlier estimates (< 200 nt; [33]). This increases the number of yeast genes with a potential to be regulated by uORFs.

Based on identification of putative functional uORFs using comparisons between mouse and man, it has been suggested that the peptides encoded by regulatory uORFs in most cases are crucial to their function [16]. Our results do not support this conclusion for yeast: a) we find no bias for synonymous vs. non-synonymous mutations in the nucleotide substitutions, in six *Saccharomyces *species, present among the uORFs most strongly predicted to be functional; b) the lack of a codon bias or strong translation start sites for conserved uORFs give no support for functional peptides to be encoded by them; c) even in a small set of otherwise conserved uORFs, we find an example of a nonsense mutation. We conclude that for the majority of functional uORFs, the encoded peptide plays no regulatory role. It should be emphasised, however, that our analysis may be biased for *GCN4*-type uORFs, with a regulatory mechanism that does not involve the encoded peptide.

We have observed a correlation between the folding energies calculated by Ringner and Krogh [21] for the 200 nt upstream of the start codon and the presence of a predicted functional uORF: 5'-UTRs with experimentally verified functional uORFs have weaker folding than average genes. The genes we predict in this work to have functional uORFs have weaker folding in this region than the average gene, but stronger than the previously recognised set. This indicates that we have selected a set of upstream regions enriched for functional uORFs (or uORFs with sequence properties similar to functional ones). Given that we find the optimal distance between a functional uORF and the start codon of the main ORF to be in the range 50 – 200 nt, it is not surprising that a correlation is found for upstream sequences of a similar length.

### Generality of the findings

We used as a starting point for this investigation the well-documented regulatory uORFs of *S. cerevisiae GCN4*. We found their evolutionary conservation to extend quite far, even beyond *Ashbya*. We did not find another example of such extensive conservation among the set of high-scoring uORFs. In fact we have identified no other uORF that is preserved in all seven *Saccharomyces *species, not even among genes with previously well-characterised functional uORFs such as *CLN3*, *YAP1 *or *YAP2*. Several components of the pathway regulating *GCN4 *expression through modulation of translation of its mRNA, *e.g*. the protein kinase Gcn2, are conserved also in plants and animals. Translational control through uORFs could potentially be a very widespread mechanism for *GCN4 *homologues, and in this respect this gene could represent a special case. Another aspect of *GCN4 *is the arrangement of 4 uORFs acting together in an intricate regulatory pattern. It is only uORF4, the most gene-proximal one, that conforms to the criterion of being located within 150 nt from the start codon of the gene. Translation of this uORF precludes translation of the main gene [6]. It is thus conceivable that the uORFs predicted to be functional in this work represent a subgroup with negative regulatory properties.

Within the group of conserved uORFs that we have examined, there is a high covariance between the property of being short (< 10 codons) of the uORF and the requirement for a certain distance (50 – 150 nucleotides) from the start codon of the main ORF. It is likely that we have defined a subset of genes containing uORFs similar to uORF4 of *GCN4*, which shares these properties. Other classes of genes with uORFs with a demonstrated functional role in translational regulation include *YAP1*, *YAP2 *and *PET111*. The uORFs of these genes are much longer (16 codons) and overlap with each other (*PET111*) and with the main ORF. It has been argued that the longer the uORF, the lower the reinitiation frequency immediately downstream of it [8]. The short uORFs in the *GCN4 *class may reflect the need for flexible reinitiation frequencies, using the uORF as a regulatory device: if the uORF is too long, then translation would be constitutively off. If so, then clearly the much longer uORFs in the other two classes should also completely repress translational reinitiation, given the narrow optimum for uORFs in the *GCN4 *class. It follows that the sequence requirements for uORFs in the other two classes have to follow different principles, and the mechanisms of action of these uORFs are presumably different from those in the *GCN4 *class. Indeed, post-termination events have been invoked to explain the action of uORFs in the *YAP2 *mRNA [28].

Our initial set of uORFs with a known functional role contained a large majority of *GCN4*-like genes, and this is a likely explanation why we have arrived at a set of rules that is biased in their favour and describes similar uORFs. Another, not mutually exclusive, explanation is that the *GCN4 *class is more homogeneous in terms of sequence requirements than other classes. A third alternative would be that *GCN4*-like uORFs are simply much more numerous in the genome, which would facilitate their detection.

### Perspective

Regulation by uORFs is in principle detectable by several experimental methods. Using fractionation of mRNA bound to several ribosomes (polysomes) or to one ribosome or ribosomal subunit (monosomes), one can observe the *GCN4 *mRNA accumulating in the monosomal fraction (characteristic of translation initiation of uORFs) under conditions of good nitrogen availability, and migrate to polysomal fractions (indicative of translation of the main ORF) under conditions of nitrogen starvation [34]. With global approaches to translational regulation, one can separate polysomal from monosomal RNA and analyse the relative abundance of all cellular mRNAs on microarrays [34-36]. In an experimental approach to enrich translationally regulated transcripts, Arava et al. [34] examined mRNAs co-sedimenting with monosomes using this approach. Using a combination of microarray experiments displaying polysomal association under several different conditions should be an efficient way to experimentally verify the predictions from this work.

## Methods

### Sequence collections and databases

From a database of 5'-UTR's from genes where the transcript start sites have been mapped [18,37], we extracted 294 5'-UTR sequences from *S. cerevisiae *and catalogued all uORFs (see electronic supplement). Genome sequences of *S. paradoxus, S. mikatae *and *S. bayanus*, as well as tabulated information about syntenic regions, were taken from Kellis et al. [12], whereas the genome sequences from *S. kudriavzevii*, *S. castellii *and *S. kluyveri *were taken from Cliften et al., 2003 [9]. Both datasets were downloaded from the Saccharomyces Genome Database (SGD [38]). 5' flanking sequences from orthologous genes were extracted from databases, and uORFs detected in them in all six reading frames using getorf with no upper or lower limits set for ORF length [39]. Intergenic sequences from the seven species were collected from the homepage of the Martha L. Bulyk laboratory at Harvard University [40].

### Alignment and visualisation of conservation of uORFs

A series of Perl scripts [41] were developed and used for performing large-scale batch analyses on the data. Upstream regions were extracted from *S. cerevisiae *and open reading frames were identified using the software getorf [39]. The candidate uORFs were assessed by an expert system (see next section) to produce a list of candidates sorted by their obtained score based on a set of rules. These candidates were aligned to the homologous regions in the six other species to verify their integrity using the AlignX module of Vector NTI Suite (Informax) and the alignment was visualised along with its DNA similarity profile (Fig. 4). Overviews of candidate uORFs in the syntenic upstream regions of the seven species were also plotted using a custom Java application ([41]; Fig. 5). We have maintained the established numbering of uORFs in the 5' to 3' direction for genes where the sequences were derived from the 5'-UTR of mRNAs (thus the well-characterised inhibitory uORF4 of *GCN4 *keeps its name), whereas numbering starts at the AUG of the main ORF and runs 3' to 5' for cases where genomic sequence was used. This is indicated in the respective tables.

### Prediction of uORF functionality using an expert system

A simple expert system was constructed to predict which uORFs were likely to affect gene expression. Attribute values describing the properties of genes and uORFs were derived from different genome sequences using a suite of programs written in Perl and Java. Attributes of interest were intergenic sequence length, the number of uORFs, the length of each uORF, and the distance in nucleotides from the uORF to the start of the main gene. These values were loaded into frames structures in an expert system shell [41].

The expert system uses a MYCIN-like certainty factor (cf) model for representing and reasoning with uncertain data and rules [42]. Cfs are values in the range -1.0 to +1.0. A value of +1.0 means that we are sure of something; a value of -1.0 means that something is definitely untrue; a value of zero means that we know nothing about whether a piece of knowledge is true or not. A set of production rules for inferring whether a uORF was likely to affect gene expression was written manually and each rule was assigned a certainty factor representing our confidence in a consequent being true if all of the antecedents are true. These rules were loaded into the expert system's rule base, and forward chaining inference was used to apply the rules to the data. If the same prediction was made for a uORF using two or more different lines of inference, then the cfs associated with these were combined as in MYCIN [42]. The resulting cf with which each uORF was predicted to affect gene expression was used to score the uORF.

As a first step, the rules were applied to training data consisting of a set of 16 genes containing uORFs, 9 of which were known to affect the translational activity (see Fig. 2, set A). The rules and their associated cfs were adjusted by hand until the expert system could distinguish between positive and negative training examples. A threshold value for the cf score for positive examples was determined by looking at the cfs inferred for known functional uORFs. The attribute values of the expert system and their certainty factor are given in additional file 5.

Having built a rule base and selected a threshold score for predicting likely functional uORFs, the expert system was used to classify all uORF-containing genes in the *S. cerevisiae *genome as likely or unlikely to be regulated by uORFs. A gene was predicted to be a "good candidate" if at least one of its uORFs was inferred to have a functional role with a cf score above the selected threshold. The highest cf value for any one of a gene's uORFs was used as the score for the gene itself.

### Calculation of synonymous and non-synonymous substitutions

The ratio of synonymous to non-synonymous substitution mutations within uORFs and in protein-coding yeast DNA was calculated. Homologous sequences from the seven species were identified using BLASTN and aligned with CLUSTALW, and differences from the *S. cerevisiae *sequence were recorded.

## Conclusion

We have identified criteria that distinguish uORFs in the yeast genome that are conserved in evolution. These are: short length of the uORF (4 – 6 nt); optimal distance from the main ORF (50 – 250 nt); greater than average distance to neighbouring uORFs; weaker than average folding energies of the 5'-UTR. These rules probably apply not to all functional uORFs in the genome, but to those similar to uORFs in *GCN4*. Evolutionary conservation of most uORFs identified extends to separation times between 20 and 100 million years ago, but *GCN4 *uORFs considerably beyond that. Using these criteria, we have identified 252 genes with uORFs that we predict to be functional, and short-listed 32 among those. We subsequently determined that the majority of these are located within transcripts. We found no bias in G/C composition near uORFs. We also found no evidence indicating that the encoded peptide of most uORFs identified in this study would play a functional role in regulation. Genes containing uORFs predicted to be functional were enriched for a function in transcriptional control, cell polarity, sporulation and development, with several genes encompassing more than one of these categories.

## Authors' contributions

MC compiled and extracted sequence data, formulated the initial rule set, assessed all alignments, and performed data analyses. DD wrote all Perl scripts and assisted in data analysis. EB formulated questions about sequence determinants of uORFs. GK implemented the rule set into expert system software. PS conceived the study and drafted the manuscript.

## Supplementary Material

Additional file 1 uORFs in the dataset by Pesole et al. [18] of verified 5'-UTRs, which we have verified to be conserved. Numbering of uORFs is 5' to 3'. Click here for file

Additional file 2 The 252 genes with conserved uORFs and with a maximal confidence factor score (0.98). Information given for each gene from left to right: Systematic name of gene, length of intergenic region in nucleotides, length of uORF in codons, position in nucleotides of uORF relative to ATG of main ORF. The first uORF listed is always the most distant from the main ATG. Genes appear in the list in no particular order.
Click here for file

Additional file 3 uORFs identified in verified 5'-UTRs by Zhang and Dietrich [19]. Numbering of uORFs is 5' to 3'. Click here for file

Additional file 4 uORFs predicted to be functional by Zhang and Dietrich [17]. Numbering of uORFs 5' to 3'. Click here for file

Additional file 5 Attribute values of the expert system and their certainty factor Click here for file

## References

[B1] Kozak M (2002). Pushing the limits of the scanning mechanism for initiation of translation. Gene.

[B2] Morris DR, Geballe AP (2000). Upstream open reading frames as regulators of mRNA translation. Mol Cell Biol.

[B3] Vilela C, McCarthy JE (2003). Regulation of fungal gene expression via short open reading frames in the mRNA 5'untranslated region. Mol Microbiol.

[B4] Delbecq P, Werner M, Feller A, Filipkowski RK, Messenguy F, Pierard A (1994). A segment of mRNA encoding the leader peptide of the CPA1 gene confers repression by arginine on a heterologous yeast gene transcript. Mol Cell Biol.

[B5] Gaba A, Jacobson A, Sachs MS (2005). Ribosome occupancy of the yeast CPA1 upstream open reading frame termination codon modulates nonsense-mediated mRNA decay. Mol Cell.

[B6] Abastado JP, Miller PF, Jackson BM, Hinnebusch AG (1991). Suppression of ribosomal reinitiation at upstream open reading frames in amino acid-starved cells forms the basis for GCN4 translational control. Mol Cell Biol.

[B7] Hinnebusch AG, Hershey JWB, Mathews MB and Sonenberg N (1996). Translational control of GCN4: gene-specific regulation
by phosphorylation of eIF2. Translational control.

[B8] Luukkonen BG, Tan W, Schwartz S (1995). Efficiency of reinitiation of translation on human immunodeficiency virus type 1 mRNAs is determined by the length of the upstream open reading frame and by intercistronic distance. J Virol.

[B9] Cliften P, Sudarsanam P, Desikan A, Fulton L, Fulton B, Majors J, Waterston R, Cohen BA, Johnston M (2003). Finding functional features in Saccharomyces genomes by phylogenetic footprinting. Science.

[B10] Cliften PF, Hillier LW, Fulton L, Graves T, Miner T, Gish WR, Waterston RH, Johnston M (2001). Surveying Saccharomyces genomes to identify functional elements by comparative DNA sequence analysis. Genome Res.

[B11] Kellis M, Patterson N, Birren B, Berger B, Lander ES (2004). Methods in comparative genomics: genome correspondence, gene identification and regulatory motif discovery. J Comput Biol.

[B12] Kellis M, Patterson N, Endrizzi M, Birren B, Lander ES (2003). Sequencing and comparison of yeast species to identify genes and regulatory elements. Nature.

[B13] Kurtzman CP, Piškur J, Sunnerhagen P and Piškur J (2006). Taxonomy and phylogenetic diversity among the yeasts. Comparative genomics using fungi as models.

[B14] Gibbs RA, Weinstock GM, Metzker ML, Muzny DM, Sodergren EJ, Scherer S, Scott G, Steffen D, Worley KC, Burch PE, Okwuonu G, Hines S, Lewis L, DeRamo C, Delgado O, Dugan-Rocha S, Miner G, Morgan M, Hawes A, Gill R, Holt RA, Adams MD, Amanatides PG, Baden-Tillson H, Barnstead M, Chin S, Evans CA, Ferriera S, Fosler C, Glodek A, Gu Z, Jennings D, Kraft CL, Nguyen T, Pfannkoch CM, Sitter C, Sutton GG, Venter JC, Woodage T, Smith D, Lee HM, Gustafson E, Cahill P, Kana A, Doucette-Stamm L, Weinstock K, Fechtel K, Weiss RB, Dunn DM, Green ED, Blakesley RW, Bouffard GG, De Jong PJ, Osoegawa K, Zhu B, Marra M, Schein J, Bosdet I, Fjell C, Jones S, Krzywinski M, Mathewson C, Siddiqui A, Wye N, McPherson J, Zhao S, Fraser CM, Shetty J, Shatsman S, Geer K, Chen Y, Abramzon S, Nierman WC, Havlak PH, Chen R, Durbin KJ, Egan A, Ren Y, Song XZ, Li B, Liu Y, Qin X, Cawley S, Cooney AJ, D'Souza LM, Martin K, Wu JQ, Gonzalez-Garay ML, Jackson AR, Kalafus KJ, McLeod MP, Milosavljevic A, Virk D, Volkov A, Wheeler DA, Zhang Z, Bailey JA, Eichler EE, Tuzun E, Birney E, Mongin E, Ureta-Vidal A, Woodwark C, Zdobnov E, Bork P, Suyama M, Torrents D, Alexandersson M, Trask BJ, Young JM, Huang H, Wang H, Xing H, Daniels S, Gietzen D, Schmidt J, Stevens K, Vitt U, Wingrove J, Camara F, Mar Alba M, Abril JF, Guigo R, Smit A, Dubchak I, Rubin EM, Couronne O, Poliakov A, Hubner N, Ganten D, Goesele C, Hummel O, Kreitler T, Lee YA, Monti J, Schulz H, Zimdahl H, Himmelbauer H, Lehrach H, Jacob HJ, Bromberg S, Gullings-Handley J, Jensen-Seaman MI, Kwitek AE, Lazar J, Pasko D, Tonellato PJ, Twigger S, Ponting CP, Duarte JM, Rice S, Goodstadt L, Beatson SA, Emes RD, Winter EE, Webber C, Brandt P, Nyakatura G, Adetobi M, Chiaromonte F, Elnitski L, Eswara P, Hardison RC, Hou M, Kolbe D, Makova K, Miller W, Nekrutenko A, Riemer C, Schwartz S, Taylor J, Yang S, Zhang Y, Lindpaintner K, Andrews TD, Caccamo M, Clamp M, Clarke L, Curwen V, Durbin R, Eyras E, Searle SM, Cooper GM, Batzoglou S, Brudno M, Sidow A, Stone EA, Payseur BA, Bourque G, Lopez-Otin C, Puente XS, Chakrabarti K, Chatterji S, Dewey C, Pachter L, Bray N, Yap VB, Caspi A, Tesler G, Pevzner PA, Haussler D, Roskin KM, Baertsch R, Clawson H, Furey TS, Hinrichs AS, Karolchik D, Kent WJ, Rosenbloom KR, Trumbower H, Weirauch M, Cooper DN, Stenson PD, Ma B, Brent M, Arumugam M, Shteynberg D, Copley RR, Taylor MS, Riethman H, Mudunuri U, Peterson J, Guyer M, Felsenfeld A, Old S, Mockrin S, Collins F, Celera (2004). Genome sequence of the Brown Norway rat yields insights into mammalian evolution. Nature.

[B15] Galagan JE, Calvo SE, Cuomo C, Ma LJ, Wortman JR, Batzoglou S, Lee SI, Basturkmen M, Spevak CC, Clutterbuck J, Kapitonov V, Jurka J, Scazzocchio C, Farman M, Butler J, Purcell S, Harris S, Braus GH, Draht O, Busch S, D'Enfert C, Bouchier C, Goldman GH, Bell-Pedersen D, Griffiths-Jones S, Doonan JH, Yu J, Vienken K, Pain A, Freitag M, Selker EU, Archer DB, Penalva MA, Oakley BR, Momany M, Tanaka T, Kumagai T, Asai K, Machida M, Nierman WC, Denning DW, Caddick M, Hynes M, Paoletti M, Fischer R, Miller B, Dyer P, Sachs MS, Osmani SA, Birren BW (2005). Sequencing of Aspergillus nidulans and comparative analysis with A. fumigatus and A. oryzae. Nature.

[B16] Crowe ML, Wang XQ, Rothnagel JA (2006). Evidence for conservation and selection of upstream open reading frames suggests probable encoding of bioactive peptides. BMC Genomics.

[B17] Zhang Z, Dietrich FS (2005). Identification and characterization of upstream open reading frames (uORF) in the 5' untranslated regions (UTR) of genes in *Saccharomyces cerevisiae*. Curr Genet.

[B18] Pesole G, Liuni S, Grillo G, Ippedico M, Larizza A, Makalowski W, Saccone C (1999). UTRdb: a specialized database of 5' and 3' untranslated regions of eukaryotic mRNAs. Nucleic Acids Res.

[B19] Zhang Z, Dietrich FS (2005). Mapping of transcription start sites in *Saccharomyces cerevisiae *using 5' SAGE. Nucleic Acids Res.

[B20] David L, Huber W, Granovskaia M, Toedling J, Palm CJ, Bofkin L, Jones T, Davis RW, Steinmetz LM (2006). A high-resolution map of transcription in the yeast genome. Proc Natl Acad Sci USA.

[B21] Ringner M, Krogh M (2005). Folding free energies of 5'-UTRs impact post-transcriptional regulation on a genomic scale in yeast. PLoS Comput Biol.

[B22] Sharp PM, Li WH (1987). The Codon Adaptation Index - a measure of directional synonymous codon usage bias, and its potential applications. Nucleic Acids Res.

[B23] Subramaniyam V, Gupta SK, Ghosh TC (2004). Shannon's uncertainty principle and gene expression levels. Curr Sci.

[B24] Meijer HA, Thomas AA (2002). Control of eukaryotic protein synthesis by upstream open reading frames in the 5'-untranslated region of an mRNA. Biochem J.

[B25] Miyasaka H (1999). The positive relationship between codon usage bias and translation initiation AUG context in *Saccharomyces cerevisiae*. Yeast.

[B26] Polymenis M, Schmidt EV (1997). Coupling of cell division to cell growth by translational control of the G1 cyclin CLN3 in yeast. Genes Dev.

[B27] Vilela C, Linz B, Rodrigues-Pousada C, McCarthy JE (1998). The yeast transcription factor genes YAP1 and YAP2 are subject to differential control at the levels of both translation and mRNA stability. Nucleic Acids Res.

[B28] Vilela C, Ramirez CV, Linz B, Rodrigues-Pousada C, McCarthy JE (1999). Post-termination ribosome interactions with the 5'UTR modulate yeast mRNA stability. EMBO J.

[B29] Wright MB, Howell EA, Gaber RF (1996). Amino acid substitutions in membrane-spanning domains of Hol1, a member of the major facilitator superfamily of transporters, confer nonselective cation uptake in Saccharomyces cerevisiae. J Bacteriol.

[B30] Green-Willms NS, Butler CA, Dunstan HM, Fox TD (2001). Pet111p, an inner membrane-bound translational activator that limits expression of the *Saccharomyces cerevisiae* mitochondrial gene COX2. J Biol Chem.

[B31] Zhou W, Edelman GM, Mauro VP (2001). Transcript leader regions of two *Saccharomyces cerevisiae* mRNAs contain internal ribosome entry sites that function in living cells. Proc Natl Acad Sci USA.

[B32] Krummeck G, Gottenof T, Rödel G (1991). AUG codons in the RNA leader sequences of the yeast PET genes CBS1 and SCO1 have no influence on translation efficiency. Curr Genet.

[B33] Hampsey M (1998). Molecular genetics of the RNA polymerase II general transcriptional machinery. Microbiol Mol Biol Rev.

[B34] Arava Y, Wang Y, Storey JD, Liu CL, Brown PO, Herschlag D (2003). Genome-wide analysis of mRNA translation profiles in *Saccharomyces cerevisiae*. Proc Natl Acad Sci USA.

[B35] Swaminathan S, Masek T, Molin C, Pospisek M, Sunnerhagen P (2006). Rck2 is required for reprogramming of ribosomes during oxidative stress. Mol Biol Cell.

[B36] Smirnova JB, Selley JN, Sanchez-Cabo F, Carroll K, Eddy AA, McCarthy JE, Hubbard SJ, Pavitt GD, Grant CM, Ashe MP (2005). Global gene expression profiling reveals widespread yet distinctive translational responses to different eukaryotic translation initiation factor 2B-targeting stress pathways. Mol Cell Biol.

[B37] UTR Resource. http://bighost.area.ba.cnr.it/BIG/UTRHome/.

[B38] Saccharomyces Genome Database. http://www.yeastgenome.org/.

[B39] Rice P, Longden I, Bleasby A (2000). EMBOSS: the European Molecular Biology Open Software Suite. Trends Genet.

[B40] Martha L. Bulyk homepage. http://thebrain.bwh.harvard.edu.

[B41] http://www.molbio.gu.se/per/web/.

[B42] Shortliffe EH, Buchanan BG (1975). A model of inexact reasoning in medicine. Mathematical Biosci.

[B43] Iizuka N, Najita L, Franzusoff A, Sarnow P (1994). Cap-dependent and cap-independent translation by internal initiation of mRNAs in cell extracts prepared from Saccharomyces cerevisiae. Mol Cell Biol.

