# Supplementary material for: Identification of putative regulatory upstream ORFs in the yeast genome using heuristics and evolutionary conservation
Source: BMC Bioinformatics. 2007 Aug 8;8:295. doi: 10.1186/1471-2105-8-295 (PMC1964767; doi:10.1186/1471-2105-8-295)
Supplement: Additional file 1 — uORFs in the dataset by Pesole et al. [18] of verified 5'-UTRs, which we have verified to be conserved. Numbering of uORFs is 5' to 3'. [file 1471-2105-8-295-S1.doc]

**Additional file 1.**

| ORF | Gene name | Length of 5’-UTR | Size (codons) of uORFs; conserved uORFs marked in bold | Position |
| --- | --- | --- | --- | --- |
| *YGL071W* | *AFT1* | 515 | **uORF2(10)**  **uORF1(3)** | -83  -473 |
| *YLR220W* | *CCC1* | 273 | uORF2(15)  **uORF1(3)** | -227  -217 |
| *YNL314W* | *DAL82* | 308 | uORF3(3)  **uORF2(10)**  **uORF1(5)** | -43  -204  -227 |
| *YEL036C* | *ANP1* | 252 | **uORF3(2)**  **uORF2(3)**  **uORF1(3)** | -194  -202  -210 |
| *YEL035C*^1^ | *UTR5* | 238 | **uORF4(18)**  **uORF3(27)**  **uORF2(16)**  **uORF1(5)** | -110  -135  -218  -232 |
| *YLR088W* | *GAA1* | 62 | **uORF1(6)** | -61 |
| *YFL014W* | *HSP12* | 609 | **uORF3(6)**  **uORF2(10)**  uORF1(13) | -428  -585  -565 |
| *YKL216W* | *URA1* | 155 | **uORF1(23)** | -116 |
| *YKR080W* | *MTD1* | 386 | **uORF3(3)**  **uORF2(3)**  uORF1(34) | -183  -243  -308 |
| *YBR080C* | *SEC18* | 85 | **uORF1(20)** | Overlaps with main ORF |
| *YDR177W* | *UBC1* | 345 | uORF3(14)  **uORF2(2)**  **uORF1(9)** | -152  -156  -140 |
| *YDL031W* | *DBP10* | 305 | uORF4(6)  uORF3(22)  **uORF3(2)**  uORF2(12) | -212  -247  -135  -130 |
| *YDL101C* | *DUN1* | 234 | uORF4(8)  **uORF3(6)**  uORF2(27)  **uORF1(4)** | -84  -117  -197  -202 |
| *YKL182W* | *FAS1* | 332 | **uORF1(6)** | -141 |
| *YKR009C* | *FOX2* | 240 | **uORF1(2)** | -171 |
| *YDL028C* | *RPK1* | 132 | **uORF1(18)** | overlaps with main ORF |

^1^For *YEL035C*, an unequivocal orthologue is only found in *S. mikatae*, where all 4 uORFs are conserved
