# Supplementary material for: Identification of putative regulatory upstream ORFs in the yeast genome using heuristics and evolutionary conservation
Source: BMC Bioinformatics. 2007 Aug 8;8:295. doi: 10.1186/1471-2105-8-295 (PMC1964767; doi:10.1186/1471-2105-8-295)
Supplement: Additional file 2 — The 252 genes with conserved uORFs and with a maximal confidence factor score (0.98). Information given for each gene from left to right: Systematic name of gene, length of intergenic region in nucleotides, length of uORF in codons, position in nucleotides of uORF relative to ATG of main ORF. The first uORF listed is always the most distant from the main ATG. Genes appear in the list in no particular order. [file 1471-2105-8-295-S2.doc]

**Additional file 2**.

YGR136W 260 **uORF(3) -236**

**uORF(3) -222**

YDR423C 157 **uORF(6) -57**

uORF(23)-23

YHR107C 267 **uORF(5) -266**

uORF(13) -231

uORF(23) -238

YKL144C 300 uORF(15) -218

**uORF(4) -198**

uORF(16) -107

YLR072W 236 **uORF(8) -183**

**uORF(6) -111**

uORF(3) -21

uORF(9) -32

YML007W 164 **uORF(7) -84**

YLR100W 222 uORF(12) -177

**uORF(3) -121**

uORF(60) -209

YAL040C 864 **uORF(4) -316**

YLR216C 255 uORF(2) -247

**uORF(3) -145**

uORF(52) -239

**uORF(9) -60**

YLR319C 256 **uORF(5) -168**

**uORF(3) -107**

uORF(27) -91

YML093W 241 uORF(11) -173

**uORF(4) -99**

YMR233W 226 **uORF(7) -194**

uORF(24) -141

uORF(11) -70

uORF(3) -38

YNL158W 265 uORF(13) -252

uORF(29) -236

**uORF(7) -136**

**uORF(3) -116**

**uORF(9) -108**

uORF(19) -95

YOL093W 260 uORF(2) -223

uORF(7) -193

uORF(16) -166

uORF(2) -108

uORF(7) -100

uORF(7) -71

YOR167C 296 uORF(32) -196

**uORF(7) -92**

uORF(21) -72

YOR254C 248 uORF(26) -207

**uORF(5) -82**

YPL029W 260 **uORF(5) -236**

uORF(34) -256

**uORF(7) -145**

**uORF(4) -106**

**uORF(8) -95**

uORF(19) -82

YPR051W 287 **uORF(5) -286**

**uORF(4) -187**

uORF(11) -74

uORF(33) -102

YPR174C 266 uORF(2) -192

**uORF(3) -181**

**uORF(5) -155**

**uORF(5) -74**

**uORF(5) -69**

YPR191W 340 uORF(2) -**228**

**uORF**(3) -223

YPR137W 342 uORF(1) -338

**uORF(4) -270**

**uORF(3) -240**

uORF(2) -210

uORF(10) -153

**uORF(5) -134**

**uORF(4) -120**

YPR113W 338 **uORF(5) -331**

**uORF(4) -273**

**uORF(2) -262**

**uORF(2) -257**

**uORF(2) -252**

**uORF(6) -247**

uORF(17) -82

YPR054W 306 uORF(6) -289

uORF(1) -27

YPR006C 340 uORF(6) -319

uORF(16) -281

**uORF(4) -206**

uORF(25) -252

uORF(11) -170

uORF(15) -131

YPL211W 346 uORF(22) -250

uORF(2) -126

**uORF(6) -121**

**uORF(3) -97**

uORF(20) -143

YPL020C 333 uORF(18) -329

**uORF(8) -169**

uORF(12) -152

YPL011C 315 uORF(7) -306

uORF(18) -256

uORF(7) -196

**uORF(2) -144**

**uORF(5) -108**

**uORF(3) -97**

YOR353C 319 **uORF(3) -265**

**uORF(5) -246**

uORF(9) -199

uORF(3) -148

uORF(27) -203

**uORF(5) -59**

uORF(23) -72

YOR251C 313 uORF(10) -275

**uORF(6) -205**

**uORF(6) -92**

uORF(5) -48

YOR166C 302 **uORF(9) -227**

**uORF(5) -76**

uORF(21) -65

YOL144W 310 uORF(13) -284

uORF(10) -232

uORF(25) -180

uORF(2) -99

**uORF(6) -89**

YNL256W 303 uORF(2) -241

**uORF(8) -249**

uORF(54) -293

**uORF(3) -66**

uORF(40) -121

YMR215W 313 **uORF(3) -289**

**uORF(4) -147**

YLR427W 315 uORF(13) -233

uORF(45) -283

**uORF(5) -146**

**uORF(6) -71**

YLR373C 330 **uORF(8) -318**

uORF(42) -262

uORF(37) -200

uORF(11) -106

uORF(24) -74

YLR243W 320 uORF(2) -250

uORF(22) -291

uORF(11) -245

uORF(12) -219

**uORF(3) -111**

YLR186W 344 **uORF(4) -317**

uORF(1) -211

uORF(2) -110

uORF(17) -126

uORF(40) -157

YLR136C 348 **uORF(4) -251**

uORF(19) -189

uORF(27) -196

uORF(43) -182

YLL051C 333 uORF(7) -294

uORF(29) -328

uORF(20) -246

uORF(1) -98

**uORF(4) -55**

uORF(6) -24

YLL036C 324 uORF(9) -304

**uORF(5) -185**

**uORF(5) -153**

uORF(16) -130

uORF(12) -63

YJR062C 346 **uORF(4) -276**

uORF(22) -281

uORF(9) -52

YIL151C 319 uORF(13) -287

uORF(9) -265

**uORF(4) -239**

YHR184W 315 uORF(2) -277

**uORF(3) -218**

**uORF(6) -199**

**uORF(6) -191**

uORF(23) -87

YHR171W 328 uORF(7) -314

uORF(1) -249

uORF(11) -242

uORF(17) -253

**uORF(5) -213**

**uORF(4) -62**

uORF(28) -130

YGL197W 322 **uORF(3) -226**

**uORF(5) -109**

YFR030W 339 uORF(9) -320

uORF(21) -171

**uORF(4) -62**

YDR198C 307 **uORF(8) -243**

uORF(18) -91

YBL031W 370 uORF(5) -310

uORF(10) -251

uORF(15) -261

uORF(12) -235

**uORF(4) -91**

uORF(2) -65

uORF(5) -39

YBR063C 400 uORF(2) -381

uORF(14) -400

uORF(39) -254

uORF(18) -169

**uORF(3) -62**

YBR103W 386 uORF(40) -339

**uORF(4) -200**

uORF(11) -210

uORF(12) -178

YCR048W 384 uORF(27) -320

uORF(1) -227

uORF(18) -268

**uORF(4) -116**

YDL051W 369 **uORF(9) -52**

uORF(48) -164

YDL176W 376 **uORF(9) -323**

**uORF(4) -294**

uORF(36) -283

uORF(46) -287

**uORF(9) -104**

YDL229W 358 **uORF(3) -274**

**uORF(9) -242**

uORF(12) -216

uORF(30) -220

uORF(18) -63

uORF(12) -38

YGL006W 387 **uORF(4) -144**

YHR064C 391 uORF(11) -380

uORF(10) -325

uORF(10) -226

uORF(21) -210

uORF(12) -145

**uORF(7) -123**

uORF(34) -103

YJL088W 384 uORF(19) -279

uORF(31) -216

**uORF(6) -83**

uORF(102) -307

YLL046C 396 **uORF(3) -322**

**uORF(8) -262**

**uORF(8) -75**

YLR088W 376 uORF(18) -327

uORF(39) -120

**uORF(6) -62**

YLR201C 357 uORF(38) -344

**uORF(7) -318**

**uORF(6) -280**

**uORF(7) -228**

uORF(10) -161

YLR443W 374 uORF(17) -279

uORF(32) -316

**uORF(9) -221**

uORF(58) -178

YNL053W 359 uORF(1) -350

uORF(1) -254

uORF(18) -276

uORF(16) -186

**uORF(4) -104**

YNL154C 393 **uORF(4) -347**

uORF(26) -354

uORF(13) -296

YNR049C 393 uORF(8) -383

uORF(2) -267

**uORF(3) -142**

YOR060C 371 **uORF(4) -309**

uORF(57) -255

uORF(17) -53

YOR061W 371 uORF(68) -349

**uORF(5) -103**

YOR112W 381 **uORF(9) -343**

uORF(12) -317

uORF(2) -274

**uORF(5) -223**

uORF(36) -115

YOR124C 388 uORF(6) -357

uORF(2) -317

uORF(11) -298

uORF(14) -256

**uORF(3) -204**

**uORF(7) -211**

YOR238W 379 **uORF(5) -374**

uORF(20) -333

uORF(20) -305

uORF(1) -218

**uORF(8) -134**

uORF(37) -199

uORF(2) -82

uORF(3) -10

YPL004C 363 **uORF(8) -146**

uORF(22) -68

YPL057C 373 uORF(25) -265

**uORF(5) -145**

YBL025W 443 uORF(30) -438

uORF(8) -365

uORF(39) -337

uORF(16) -218

**uORF(9) -146**

uORF(25) -171

**uORF(9) -115**

uORF(21) -85

YCL027W 436 uORF(33) -433

uORF(16) -298

uORF(30) -332

uORF(2) -239

**uORF(5) -192**

**uORF(4) -178**

**uORF(3) -121**

**uORF(4) -92**

YCL057W 443 **uORF(9) -368**

**uORF(6) -193**

uORF(12) -176

uORF(18) -157

uORF(47) -207

uORF(13) -42

YDL014W 430 uORF(12) -410

uORF(16) -277

uORF(32) -282

**uORF(7)** -191

YER118C 441 **uORF(8) -212**

YGL248W 441 **uORF(5) -344**

uORF(17) -375

**uORF(5) -258**

uORF(19) -248

**uORF(3) -192**

uORF(28) -175

YLR060W 432 uORF(1) -285

uORF(24) -263

**uORF(8) -87**

uORF(19) -101

YOL113W 401 uORF(16) -401

uORF(13) -351

**uORF(9) -282**

**uORF(9) -208**

uORF(19) -182

uORF(13) -103

uORF(25) -77

YOR308C 438 **uORF(5) -436**

uORF(12) -357

**uORF(3) -234**

uORF(20) -67

uORF(23) -72

YPR016C 417 **uORF(4) -340**

uORF(1) -281

uORF(20) -247

uORF(20) -243

uORF(1) -135

YBR040W 458 **uORF(6) -407**

uORF(27) -387

uORF(11) -213

uORF(27) -224

uORF(40) -175

uORF(29) -89

YDL146W 489 uORF(35) -421

uORF(17) -362

**uORF(3) -302**

uORF(53) -369

**uORF(6) -211**

**uORF(3) -53**

YKL093W 499 uORF(14) -434

uORF(22) -292

uORF(11) -191

uORF(14) -165

**uORF(6) -71**

YLR009W 454 **uORF(4) -430**

uORF(11) -320

**uORF(3) -220**

YLR083C 495 **uORF(3) -457**

**uORF(9) -331**

uORF(27) -372

**uORF(9) -222**

uORF(10) -187

uORF(35) -215

YLR220W 488 uORF(1) -393

uORF(15) -228

**uORF(3) -218**

YMR010W 459 **uORF(9) -432**

**uORF(5) -378**

**uORF(9) -229**

uORF(44) -279

YMR014W 477 uORF(20) -411

uORF(1) -312

**uORF(4) -307**

uORF(13) -267

uORF(20) -136

uORF(12) -73

uORF(25) -77

YMR144W 459 **uORF(4) -365**

uORF(22) -309

**uORF(4) -232**

uORF(40) -293

**uORF(8) -190**

YNL229C 458 uORF(11) -385

**uORF(5) -285**

uORF(1) -242

YOR231W 488 uORF(37) -483

**uORF(4) -361**

uORF(13) -365

uORF(42) -303

**uORF(9) -72**

YDR253C 548 uORF(17) -478

uORF(13) -382

uORF(18) -363

**uORF(8) -119**

**uORF(5) -106**

**uORF(5) -71**

uORF(10) -78

YDR346C 519 **uORF(4) -468**

uORF(10) -388

uORF(13) -381

**uORF(4) -226**

uORF(43) -251

YDR466W 547 uORF(10) -484

uORF(2) -455

uORF(2) -450

**uORF(6) -445**

**uORF(8) -299**

uORF(22) -309

**uORF(9) -214**

uORF(28) -254

uORF(13) -171

uORF(2) -8

YDR480W 525 **uORF(5) -467**

**uORF(3) -261**

uORF(15) -253

uORF(18) -196

**uORF(7) -140**

uORF(15) -87

uORF(4) -50

YEL013W 541 uORF(2) -467

**uORF(7) -459**

uORF(10) -439

**uORF(3) -364**

**uORF(5) -306**

YEL061C 509 uORF(12) -342

uORF(17) -353

uORF(11) -249

**uORF(4) -110**

uORF(38) -115

YIL002C 509 **uORF(8) -505**

uORF(25) -468

uORF(13) -338

uORF(28) -245

uORF(8) -27

YJL139C 505 uORF(20) -448

**uORF(4) -352**

uORF(31) -287

**uORF(8) -55**

YJR036C 545 uORF(21) -441

uORF(19) -379

**uORF(4) -172**

uORF(21) -215

uORF(31) -110

YLR301W 524 **uORF(8) -487**

**uORF(4) -359**

uORF(1) -318

uORF(11) -133

YLR312C 550 uORF(37) -441

**uORF(9) -207**

uORF(13) -202

uORF(22) -148

uORF(33) -108

uORF(15) -50

YML106W 504 uORF(20) -465

**uORF(4) -410**

uORF(7) -46

YMR126C 540 uORF(1) -527

uORF(23) -510

uORF(33) -517

uORF(20) -416

uORF(16) -297

**uORF(5) -200**

YOL041C 503 **uORF(4) -454**

uORF(2) -370

**uORF(9) -361**

uORF(13) -282

uORF(32) -266

YDL078C 583 uORF(33) -436

**uORF(3) -287**

uORF(52) -348

uORF(45) -266

YDR308C 599 uORF(88) -585

**uORF(5) -207**

uORF(26) -241

uORF(64) -233

uORF(4) -14

YJR104C 567 **uORF(3) -507**

**uORF(4) -489**

uORF(1) -401

uORF(47) -484

**uORF(6) -344**

uORF(10) -274

uORF(32) -245

uORF(24) -93

uORF(23) -79

YLR022C 572 uORF(93) -568

uORF(2) -201

**uORF(3) -175**

uORF(17) -167

**uORF(7) -111**

uORF(2) -68

YLR293C 569 uORF(44) -406

**uORF(4) -272**

**uORF(9) -261**

uORF(29) -134

YMR257C 589 **uORF(4) -562**

**uORF(5) -409**

uORF(30) -395

**uORF(10) -109**

uORF(29) -90

YNL218W 575 **uORF(3) -492**

**uORF(8) -313**

uORF(59) -279

YNR032W 577 uORF(23) -527

**uORF(8) -450**

**uORF(3) -185**

**uORF(4) -167**

**uORF(9) -132**

YPL106C 588 uORF(2) -536

**uORF(8) -442**

**uORF(5) -399**

uORF(2) -385

**uORF(6) -304**

uORF(5) -214

uORF(15) -191

YCR004C 628 **uORF(3) -580**

uORF(21) -520

uORF(12) -445

**uORF(8) -361**

YDR145W 637 **uORF(6) -484**

**uORF(3) -409**

uORF(37) -477

uORF(10) -345

uORF(2) -291

**uORF(3) -282**

uORF(12) -268

**uORF(6) -243**

uORF(19) -254

uORF(2) -137

uORF(29) -198

uORF(44) -226

**uORF(6) -105**

YER090W 609 uORF(2) -574

uORF(15) -551

**uORF(7) -500**

uORF(11) -473

**uORF(7) -259**

uORF(11) -94

uORF(24) -108

YGL032C 634 uORF(9) -607

uORF(21) -629

**uORF(5) -470**

uORF(11) -452

uORF(37) -416

uORF(1) -298

**uORF(3) -253**

uORF(13) -109

uORF(22) -71

YGR089W 638 **uORF(4) -529**

uORF(34) -464

uORF(16) -397

uORF(16) -338

uORF(4) -14

YHL015W 630 uORF(24) -354

uORF(58) -409

**uORF(6) -218**

**uORF(6) -201**

uORF(2) -175

**uORF(9) -82**

YHR071W 624 uORF(12) -554

**uORF(5) -501**

**uORF(6) -173**

uORF(13) -134

YJL190C 631 **uORF(5) -576**

uORF(37) -580

**uORF(6) -303**

**uORF(7) -170**

uORF(19) -132

**uORF(5) -56**

YLR150W 644 **uORF(7) -440**

uORF(10) -405

uORF(40) -394

**uORF(5) -226**

YNR003C 646 uORF(11) -620

**uORF(5) -466**

uORF(1) -370

uORF(1) -245

uORF(20) -226

uORF(12) -183

**uORF(3) -74**

uORF(10) -61

YOL137W 271 uORF(10) -214

uORF(5) -165

uORF(3) -107

uORF(2) -75

YPL086C 621 **uORF(3) -561**

uORF(27) -604

uORF(20) -473

**uORF(9) -396**

uORF(34) -389

**uORF(3) -242**

**uORF(5) -115**

uORF(12) -129

YPL254W 603 **uORF(3) -550**

uORF(17) -584

**uORF(7) -453**

**uORF(4) -419**

uORF(1) -281

YEL026W 692 uORF(13) -630

**uORF(4) -475**

uORF(40) -494

uORF(30) -426

**uORF(3) -206**

uORF(2) -198

uORF(2) -193

YER092W 688 uORF(18) -681

**uORF(5) -624**

uORF(53) -634

uORF(16) -397

uORF(17) -350

**uORF(3) -166**

uORF(15) -155

uORF(20) -111

uORF(41) -145

uORF(15) -48

YFL027C 668 uORF(12) -653

uORF(1) -505

**uORF(9) -481**

uORF(23) -442

uORF(29) -390

uORF(22) -222

uORF(61) -301

uORF(2) -33

YFL030W 684 **uORF(3) -527**

uORF(10) -295

uORF(37) -299

**uORF(8) -89**

uORF(58) -189

YGR285C 660 **uORF(7) -650**

uORF(42) -535

uORF(28) -449

**uORF(4) -356**

**uORF(4) -303**

uORF(42) -251

**uORF(3) -52**

uORF(40) -126

YHR052W 697 **uORF(8) -533**

uORF(18) -431

uORF(28) -375

**uORF(7) -301**

uORF(1) -195

uORF(27) -184

YKL113C 695 **uORF(3) -677**

uORF(13) -581

**uORF(8) -556**

**uORF(4) -530**

uORF(3) -414

uORF(16) -327

**uORF(4) -152**

uORF(20) -111

uORF(29) -89

YLR028C 661 uORF(13) -653

**uORF(8) -612**

uORF(78) -582

uORF(22) -395

uORF(23) -300

YLR108C 657 uORF(12) -543

uORF(1) -465

uORF(62) -586

**uORF(9) -401**

**uORF(3) -290**

uORF(2) -193

uORF(30) -263

YLR109W 657 uORF(32) -652

uORF(42) -505

**uORF(7) -370**

**uORF(7) -274**

**uORF(8) -177**

uORF(1) -143

YLR196W 664 uORF(11) -635

**uORF(4) -551**

uORF(23) -514

uORF(27) -320

uORF(22) -215

uORF(2) -133

uORF(11) -123

YLR413W 683 **uORF(3) -678**

**uORF(4) -640**

uORF(1) -593

uORF(20) -553

uORF(17) -522

**uORF(3) -367**

**uORF(3) -55**

uORF(4) -50

YML035C 665 **uORF(6) -599**

uORF(12) -576

**uORF(8) -510**

uORF(2) -373

**uORF(6) -289**

YMR063W 698 uORF(7) -695

uORF(26) -605

uORF(19) -491

uORF(29) -396

**uORF(5) -310**

**uORF(5) -293**

uORF(11) -303

uORF(2) -234

**uORF(6) -229**

**uORF(6) -212**

**uORF(5) -111**

uORF(20) -91

YMR146C 675 uORF(9) -664

uORF(29) -653

uORF(30) -399

uORF(13) -316

uORF(26) -217

**uORF(8) -85**

YNL064C 661 uORF(21) -661

uORF(11) -482

uORF(1) -440

**uORF(4) -328**

**uORF(4) -300**

**uORF(4) -230**

YBL064C 730 **uORF(9) -671**

uORF(22) -658

uORF(16) -570

uORF(63) -557

uORF(16) -397

uORF(35) -328

uORF(12) -171

uORF(75) -293

uORF(23) -108

uORF(2) -35

YDL004W 720 uORF(16) -593

**uORF(3)** -457

uORF(26) -462

uORF(55) -473

uORF(1) -279

uORF(24) -173

**uORF(3)** -105

uORF(30) -93

YHR172W 730 uORF(21) -630

uORF(1) -538

uORF(10) -521

uORF(17) -424

uORF(41) -450

uORF(21) -328

uORF(31) -299

**uORF(3) -171**

YKL004W 703 uORF(16) -546

**uORF(5) -499**

uORF(2) -444

YKL064W 707 **uORF(3) -634**

**uORF(7) -537**

uORF(1) -495

uORF(12) -512

**uORF(8) -367**

uORF(29) -334

uORF(1) -225

**uORF(9) -102**

YKL179C 712 uORF(1) -615

uORF(5) -570

uORF(40) -607

uORF(44) -289

**uORF(5) -151**

**uORF(7) -137**

**uORF(5) -117**

**uORF(6) -98**

**uORF(5) -81**

YNL014W 708 **uORF(3) -544**

uORF(24) -464

**uORF(5) -279**

uORF(27) -337

uORF(39) -179

YNL042W 730 **uORF(4) -686**

uORF(40) -723

uORF(11) -466

uORF(2) -395

**uORF(4) -390**

**uORF(6) -197**

uORF(12) -38

YNL125C 725 uORF(1) -511

uORF(83) -608

uORF(31) -233

**uORF(5) -137**

YNL220W 718 uORF(8) -678

uORF(41) -707

uORF(41) -630

**uORF(3) -508**

uORF(24) -500

uORF(17) -468

uORF(11) -373

uORF(17) -319

YAL003W 743 uORF(6) -685

uORF(25) -543

uORF(28) -429

uORF(21) -352

**uORF(7) -53**

YBL055C 760 uORF(3) -701

uORF(18) -676

uORF(5) -623

uORF(21) -411

**uORF(3) -85**

uORF(15) -107

YDR085C 778 **uORF(8) -706**

**uORF(5) -655**

uORF(41) -561

uORF(17) -439

uORF(18) -340

uORF(34) -384

uORF(45) -259

YER167W 757 **uORF(5) -755**

**uORF(9) -734**

uORF(13) -642

**uORF(3) -523**

**uORF(3) -499**

**uORF(9) -427**

**uORF(5) -363**

**uORF(7) -245**

YGL071W 758 **uORF(7) -710**

uORF(19) -652

**uORF(4) -603**

uORF(14) -592

**uORF(3) -473**

uORF(10) -83

YHR204W 789 **uORF(6) -742**

**uORF(4) -635**

uORF(25) -611

**uORF(9) -479**

**uORF(3) -331**

uORF(21) -368

uORF(12) -318

uORF(39) -310

**uORF(9) -206**

uORF(21) -237

uORF(2) -73

**uORF(4) -65**

uORF(15) -48

uORF(8) -29

YKL051W 766 uORF(23) -745

uORF(44) -674

uORF(24) -499

uORF(37) -428

**uORF(9) -292**

uORF(19) -288

uORF(20) -218

YKL104C 765 uORF(19) -675

uORF(14) -539

**uORF(8) -498**

uORF(25) -523

uORF(17) -331

YLR055C 781 uORF(1) -689

uORF(19) -736

uORF(3) -676

uORF(10) -680

uORF(25) -664

uORF(37) -541

uORF(34) -415

uORF(27) -304

**uORF(8) -224**

uORF(2) -184

uORF(59) -333

uORF(22) -188

**uORF(7) -79**

YLR325C 795 uORF(37) -790

uORF(15) -684

uORF(2) -627

**uORF(3) -622**

uORF(29) -647

uORF(12) -497

uORF(1) -386

uORF(11) -292

uORF(36) -255

uORF(36) -176

YNL094W 771 **uORF(3) -768**

**uORF(7) -666**

**uORF(4) -618**

uORF(22) -575

uORF(50) -553

uORF(24) -423

uORF(30) -282

**uORF(3) -155**

YOR271C 771 uORF(14) -675

uORF(28) -697

uORF(17) -485

uORF(32) -445

uORF(52) -489

**uORF(3) -329**

uORF(15) -308

uORF(11) -292

**uORF(4) -253**

uORF(32) -228

YBR077C 831 uORF(10) -744

**uORF(3) -715**

**uORF(3) -657**

uORF(76) -597

uORF(53) -350

uORF(2) -186

**uORF(5) -177**

uORF(18) -150

uORF(55) -250

uORF(2) -83

YDR123C 847 uORF(38) -822

uORF(34) -722

uORF(57) -663

uORF(12) -477

uORF(2) -424

**uORF(4) -379**

uORF(36) -368

uORF(31) -330

uORF(1) -144

uORF(6) -21

YDR124W 847 uORF(19) -829

uORF(14) -801

uORF(10) -773

**uORF(3) -744**

**uORF(3) -736**

uORF(13) -706

uORF(29) -668

uORF(13) -589

uORF(22) -532

uORF(58) -393

uORF(23) -278

uORF(15) -119

uORF(10) -49

uORF(20) -68

uORF(18) -57

YDR224C 818 uORF(7) -806

uORF(21) -802

uORF(46) -701

uORF(44) -528

**uORF(4) -394**

uORF(27) -333

uORF(36) -253

**uORF(7) -143**

uORF(30) -110

YDR225W 818 uORF(10) -807

uORF(18) -815

**uORF(7) -746**

uORF(24) -693

uORF(33) -704

uORF(12) -380

uORF(35) -305

YEL047C 832 uORF(36) -815

uORF(22) -691

**uORF(9) -620**

**uORF(7) -598**

uORF(46) -708

**uORF(8) -557**

**uORF(7) -467**

uORF(30) -366

uORF(30) -358

uORF(29) -281

uORF(27) -191

YER129W 811 uORF(2) -781

**uORF(3) -767**

**uORF(5) -695**

uORF(22) -732

**uORF(8) -588**

**uORF(7) -551**

**uORF(4) -341**

uORF(2) -117

YHR009C 840 **uORF(4) -826**

uORF(27) -741

uORF(11) -613

uORF(1) -448

uORF(1) -440

uORF(2) -426

uORF(19) -303

uORF(44) -362

uORF(48) -247

YIR027C 826 uORF(1) -795

uORF(8) -812

uORF(11) -789

**uORF(3) -697**

uORF(16) -679

**uORF(8) -614**

uORF(22) -448

uORF(13) -305

uORF(2) -257

**uORF(3) -249**

uORF(28) -238

**uORF(3) -155**

uORF(19) -133

YKR004C 843 uORF(11) -801

uORF(14) -747

uORF(74) -841

**uORF(4) -515**

uORF(24) -438

uORF(46) -454

**uORF(4) -295**

uORF(80) -359

uORF(1) -40

uORF(5) -17

YLR047C 825 uORF(40) -691

uORF(12) -437

uORF(20) -429

uORF(37) -433

uORF(10) -314

uORF(13) -318

**uORF(3) -230**

YLR105C 826 uORF(15) -766

uORF(36) -665

uORF(20) -588

**uORF(8) -505**

**uORF(4) -328**

**uORF(7) -317**

uORF(1) -290

**uORF(9) -279**

uORF(42) -310

uORF(37) -230

uORF(10) -68

YLR353W 813 uORF(15) -758

uORF(19) -745

uORF(12) -686

**uORF(6) -644**

**uORF(6) -549**

**uORF(7) -513**

uORF(2) -8

YPL103C 803 uORF(8) -766

uORF(6) -712

uORF(12) -673

uORF(66) -798

uORF(13) -533

uORF(14) -444

**uORF(4) -272**

uORF(20) -304

uORF(18) -282

uORF(13) -242

**uORF(8) -201**

**uORF(8) -104**

uORF(2) -59

uORF(6) -20

YPL262W 830 uORF(28) -779

uORF(14) -700

uORF(13) -656

**uORF(8) -511**

uORF(14) -437

**uORF(8) -403**

uORF(24) -386

uORF(57) -453

**uORF(6) -283**

YPR026W 821 uORF(42) -669

uORF(44) -646

**uORF(4) -65**

YAL053W 877 **uORF(3) -864**

**uORF(7) -842**

uORF(29) -767

uORF(40) -674

uORF(35) -589

uORF(45) -510

uORF(10) -386

uORF(45) -478

uORF(12) -351

**uORF(7) -199**

**uORF(6) -158**

uORF(16) -154

YBL016W 868 uORF(31) -860

uORF(2) -723

uORF(30) -655

uORF(1) -309

uORF(40) -304

**uORF(8) -181**

YBL052C 879 uORF(15) -860

uORF(7) -813

uORF(24) -705

**uORF(5) -634**

**uORF(5) -620**

uORF(11) -624

**uORF(4) -468**

uORF(11) -445

uORF(53) -479

**uORF(9) -321**

uORF(16) -226

YBR126C 877 uORF(18) -771

uORF(22) -779

uORF(40) -652

uORF(46) -500

**uORF(5) -229**

**uORF(3) -182**

uORF(12) -198

YBR203W 859 **uORF(6) -732**

uORF(24) -638

uORF(49) -648

**uORF(7) -459**

uORF(1) -321

YCR011C 873 **uORF(5)** -762

uORF(38) -832

uORF(1) -715

uORF(15) -737

**uORF(3) -543**

uORF(21) -325

**uORF(6)** -263

uORF(2) -205

**uORF(7)** -158

uORF(37) -213

YDL205C 860 uORF(20) -856

**uORF(7) -691**

uORF(20) -600

uORF(38) -483

**uORF(4) -335**

uORF(14) -324

**uORF(3) -238**

**uORF(8) -130**

YDR146C 889 uORF(81) -888

uORF(18) -653

uORF(19) -596

**uORF(3) -483**

uORF(22) -344

uORF(15) -184

uORF(35) -230

YDR435C 852 uORF(43) -836

uORF(37) -711

uORF(11) -613

uORF(78) -668

**uORF(7) -445**

uORF(22) -395

uORF(13) -223

**uORF(6) -159**

uORF(53) -236

**uORF(9) -62**

uORF(8) -27

YDR436W 852 uORF(42) -819

**uORF(3) -691**

uORF(12) -589

uORF(31) -569

uORF(71) -484

uORF(96) -420

YFR028C 869 **uORF(3) -839**

uORF(37) -835

uORF(2) -692

uORF(46) -771

**uORF(8) -494**

**uORF(3) -443**

uORF(14) -406

**uORF(5) -273**

**uORF(3) -164**

uORF(26) -225

uORF(26) -95

YHR206W 895 uORF(1) -746

**uORF(6) -733**

uORF(12) -737

uORF(14) -702

**uORF(7) -493**

uORF(2) -424

**uORF(6) -386**

uORF(15) -396

uORF(1) -297

uORF(62) -346

YOL001W 885 **uORF(8) -880**

**uORF(4) -864**

uORF(4) -774

uORF(15) -591

uORF(22) -542

uORF(15) -499

uORF(18) -349

uORF(30) -288

YGL124C 903 **uORF(7) -861**

**uORF(3) -691**

uORF(16) -608

uORF(25) -616

**uORF(4) -521**

**uORF(3) -458**

uORF(24) -336

**uORF(7) -219**

uORF(25) -146

YGR221C 931 **uORF(4) -916**

uORF(20) -902

uORF(15) -816

**uORF(8) -741**

uORF(16) -663

uORF(54) -598

uORF(18) -430

uORF(25) -422

**uORF(4) -306**

**uORF(3) -254**

uORF(13) -195

uORF(26) -185

uORF(2) -17

YNL004W 904 uORF(22) -874

uORF(12) -840

uORF(11) -805

**uORF(5) -551**

**uORF(3) -525**

**uORF(6) -477**

**uORF(3) -456**

uORF(20) -487

**uORF(3) -386**

uORF(24) -418

uORF(10) -347

uORF(1) -260

uORF(31) -337

**uORF(3) -227**

**uORF(6) -216**

uORF(2) -149

**uORF(7) -141**

**uORF(8) -111**

uORF(44) -199

uORF(7) -65

YDL021W 953 uORF(10) -872

**uORF(3) -779**

**uORF(4) -620**

uORF(50) -624

uORF(37) -560

uORF(30) -520

uORF(1) -366

uORF(19) -376

uORF(22) -229

YER158C 952 uORF(1) -939

uORF(44) -922

uORF(20) -818

**uORF(7) -759**

**uORF(9) -697**

**uORF(7) -372**

**uORF(3) -347**

YIL031W 964 **uORF(8) -918**

uORF(1) -877

uORF(14) -899

uORF(2) -684

uORF(25) -698

uORF(36) -666

**uORF(7) -519**

**uORF(4) -475**

uORF(16) -330

uORF(24) -277

uORF(9) -30

YLR224W 974 **uORF(5) -884**

uORF(17) -870

uORF(1) -768

uORF(26) -836

uORF(14) -793

uORF(20) -691

uORF(16) -672

uORF(2) -552

uORF(1) -458

uORF(36) -547

uORF(15) -419

uORF(13) -42

YLR229C 971 uORF(2) -926

**uORF(7) -770**

**uORF(3) -648**

uORF(13) -369

uORF(19) -302

uORF(60) -246

YNL282W 992 uORF(27) -978

uORF(24) -884

**uORF(9) -810**

uORF(2) -730

uORF(40) -710

**uORF(7) -563**

**uORF(8) -521**

**uORF(9) -487**

uORF(59) -444

uORF(5) -247

uORF(26) -190

uORF(47) -242

uORF(19) -66

YPL075W 969 uORF(5) -956

uORF(2) -706

uORF(11) -698

uORF(90) -834

uORF(1) -499

**uORF(4) -480**

**uORF(6) -377**

**uORF(3) -309**

**uORF(8) -296**

**uORF(5) -279**

**uORF(1) -184**

**uORF(6) -102**

YAL047C 172 **uORF(5) -94**

YBL067C 1023 **uORF(4) -924**

**uORF(9) -837**

**uORF(7) -811**

uORF(1) -650

uORF(18) -632

**uORF(6) -574**

uORF(11) -502

uORF(53) -470

uORF(15) -300

YBR025C 1012 **uORF(8) -959**

**uORF(6) -907**

**uORF(8) -856**

uORF(38) -816

uORF(16) -632

uORF(11) -601

uORF(28) -548

**uORF(7) -324**

**uORF(6) -287**

uORF(2) -177

uORF(31) -194

YBR054W 2350 uORF(6) -2325

uORF(2) -2272

uORF(6) -2280

**uORF(3) -2184**

uORF(18) -2114

uORF(25) -2110

uORF(42) -1942

uORF(15) -1807

**uORF(5) -1750**

uORF(10) -1739

uORF(22) -1719

uORF(7) -1558

uORF(8) -1491

uORF(17) -1322

uORF(6) -1251

uORF(49) -1209

uORF(1) -1034

uORF(38) -1120

uORF(1) -992

uORF(1) -947

uORF(13) -894

**uORF(9) -856**

uORF(31) -848

**uORF(5) -690**

uORF(12) -686

**uORF(9) -667**

**uORF(7) -629**

uORF(10) -508

uORF(37) -521

uORF(24) -392

YCR015C 1464 uORF(9) -1449

**uORF(4) -1408**

uORF(11) -1318

uORF(12) -1207

uORF(19) -1172

uORF(12) -1119

uORF(12) -1091

**uORF(4) -1009**

uORF(13) -942

**uORF(3) -836**

**uORF(7) -821**

uORF(1) -771

**uORF(5) -758**

uORF(1) -715

**uORF(5) -619**

uORF(104) -705

uORF(15) -431

**uORF(4) -354**

uORF(14) -370

uORF(1) -316

**uORF(4) -172**

uORF(1) -124

uORF(20) -63

YDL139C 1305 uORF(2) -1251

uORF(2) -1198

uORF(20) -1174

uORF(18) -1163

uORF(36) -1128

uORF(23) -1079

uORF(32) -1090

uORF(17) -920

**uORF(5) -863**

uORF(2) -819

uORF(14) -769

uORF(41) -782

uORF(15) -684

**uORF(7) -476**

uORF(17) -430

**uORF(4) -383**

uORF(30) -444

uORF(19) -344

**uORF(9) -269**

uORF(22) -264

uORF(12) -143

uORF(11) -108

uORF(24) -73

YDR420W 1195 uORF(8) -1173

uORF(16) -1120

uORF(9) -1001

uORF(48) -1063

uORF(1) -871

**uORF(4) -859**

uORF(28) -914

**uORF(8) -802**

**uORF(7) -762**

uORF(37) -717

uORF(37) -546

uORF(3) -11

YEL007W 2128 uORF(2) -2064

**uORF(3) -2052**

uORF(14) -1954

uORF(9) -1761

uORF(7) -1676

uORF(28) -1627

uORF(17) -1384

uORF(25) -1295

uORF(9) -1175

uORF(25) -1189

uORF(1) -1075

**uORF(3) -1034**

uORF(126) -1380

uORF(2) -833

uORF(48) -850

uORF(96) -867

uORF(44) -626

**uORF(8) -474**

YEL009C 2128 uORF(8) -2094

uORF(19) -2074

uORF(13) -2012

uORF(23) -1834

uORF(2) -1746

uORF(7) -1693

uORF(4) -1462

uORF(4) -1366

uORF(16) -1202

uORF(30) -1056

uORF(47) -1024

uORF(22) -942

uORF(19) -850

uORF(9) -757

uORF(6) -731

**uORF(3) -362**

**uORF(2) -294**

**uORF(3) -177**

**uORF(3) -152**

YER012W 1823 uORF(1) -1729

uORF(17) -1721

uORF(33) -1626

uORF(6) -1464

uORF(7) -1429

uORF(5) -1419

uORF(13) -1412

uORF(11) -1395

**uORF(5) -1298**

**uORF(4) -1259**

**uORF(5) -1145**

uORF(23) -1191

uORF(48) -1255

uORF(26) -1020

**uORF(3) -943**

uORF(41) -780

**uORF(6) -655**

**uORF(5) -624**

uORF(1) -404

**uORF(5) -285**

uORF(23) -246

uORF(32) -217

YER098W 2230 **uORF(4) -2033**

uORF(9) -1993

uORF(23) -2018

uORF(17) -1896

uORF(44) -1812

uORF(19) -1555

uORF(10) -1515

**uORF(4) -1438**

uORF(2) -1246

uORF(10) -1210

uORF(1) -1125

uORF(18) -1151

**uORF(3) -1075**

uORF(13) -998

uORF(1) -957

**uORF(4) -932**

**uORF(3) -916**

uORF(1) -798

uORF(23) -766

uORF(40) -656

uORF(47) -437

uORF(2) -272

uORF(94) -433

**uORF(5) -106**

uORF(108) -327

uORF(2) -8

YER109C 1148 uORF(1) -1047

**uORF(4) -988**

uORF(15) -984

uORF(1) -932

uORF(30) -961

uORF(13) -872

uORF(28) -815

uORF(21) -732

**uORF(9) -692**

uORF(1) -635

uORF(1) -607

uORF(28) -618

uORF(15) -503

uORF(14) -472

uORF(13) -421

uORF(26) -393

**uORF(4) -235**

**uORF(3) -224**

uORF(72) -309

YER130C 1506 uORF(8) -1365

uORF(33) -1415

uORF(7) -1130

uORF(3) -1033

uORF(14) -989

uORF(13) -943

uORF(26) -905

uORF(70) -963

uORF(30) -736

uORF(30) -699

**uORF(9) -476**

**uORF(9) -379**

uORF(27) -329

uORF(41) -271

**uORF(3) -145**

**uORF(7) -119**

uORF(10) -99

YER172C 279 uORF(31) -214

uORF(8) -128

uORF(6) -114

YGL162W 1735 **uORF(3) -1716**

uORF(8) -1412

uORF(26) -1428

**uORF(4) -1354**

**uORF(4) -1324**

uORF(11) -1282

uORF(11) -1170

uORF(1) -1134

uORF(75) -1160

uORF(23) -955

uORF(51) -887

uORF(56) -728

uORF(62) -604

uORF(19) -465

uORF(10) -276

uORF(11) -95

uORF(4) -14

YGR148C 1252 uORF(12) -1236

uORF(11) -1186

uORF(40) -1220

uORF(11) -1089

uORF(17) -1031

uORF(13) -1006

uORF(10) -963

uORF(18) -937

uORF(13) -879

uORF(1) -795

uORF(1) -617

uORF(2) -585

**uORF(5) -398**

**uORF(4) -377**

**uORF(9) -307**

uORF(43) -233

uORF(14) -53

YGR214 1110 uORF(11) -1047

uORF(12) -1015

**uORF(8) -980**

uORF(19) -957

uORF(33) -770

**uORF(4) -665**

uORF(14) -658

uORF(13) -581

uORF(16) -558

**uORF(7) -511**

**uORF(7) -444**

**uORF(6) -301**

uORF(44) -381

uORF(11) -208

uORF(25) -246

uORF(1) -85

uORF(34) -104

YGR254W 1787 uORF(8) -1756

uORF(13) -1672

uORF(9) -1656

**uORF(4) -1535**

uORF(27) -1575

uORF(14) -1519

uORF(29) -1420

uORF(2) -1312

uORF(8) -1307

uORF(40) -1263

uORF(10) -1166

**uORF(4) -1105**

uORF(13) -1115

uORF(21) -790

**uORF(4) -708**

uORF(15) -712

uORF(11) -664

uORF(63) -743

uORF(25) -552

**uORF(8) -344**

uORF(23) -290

uORF(13) -206

**uORF(9) -168**

uORF(23) -129

YHL034C 1178**uORF(5) -1171**

uORF(8) -1102

uORF(6) -1045

uORF(2) -982

uORF(13) -952

**uORF(6) -896**

uORF(20) -802

uORF(1) -661

**uORF(8) -616**

uORF(65) -716

uORF(13) -538

**uORF(5) -329**

uORF(12) -315

uORF(34) -260

YHR092C 2102 uORF(24) -2086

uORF(2) -1900

uORF(2) -1890

uORF(36) -1982

**uORF(4) -1829**

uORF(11) -1843

uORF(9) -1760

uORF(11) -1693

uORF(18) -1670

uORF(13) -1569

uORF(28) -1580

uORF(30) -1497

uORF(2) -1404

uORF(21) -1457

uORF(46) -1489

uORF(2) -1220

uORF(20) -1162

uORF(12) -1071

**uORF(4) -1032**

**uORF(7) -995**

**uORF(5) -937**

uORF(13) -851

uORF(63) -951

uORF(3) -607

uORF(50) -690

uORF(35) -483

uORF(24) -379

**uORF(5) -257**

uORF(11) -180

uORF(20) -92

YHR094C 2044 uORF(8) -1913

uORF(61) -2004

uORF(19) -1846

uORF(32) -1614

uORF(99) -1471

uORF(33) -994

uORF(1) -803

uORF(19) -763

uORF(37) -744

**uORF(5) -512**

**uORF(7) -432**

**uORF(6) -409**

uORF(15) -382

**uORF(9) -338**

**uORF(6) -259**

uORF(32) -290

**uORF(3) -101**

uORF(16) -69

YHR140W 1373 uORF(1) -1352

uORF(12) -1319

uORF(13) -1252

uORF(1) -1121

uORF(31) -1204

**uORF(5) -985**

uORF(7) -868

uORF(16) -861

**uORF(5) -817**

uORF(15) -810

uORF(28) -749

**uORF(6) -660**

uORF(55) -763

uORF(2) -596

uORF(10) -586

uORF(10) -581

**uORF(6) -494**

uORF(40) -532

uORF(20) -453

**uORF(9) -404**

uORF(24) -397

**uORF(3) -280**

uORF(27) -233

**uORF(8) -150**

uORF(13) -86

YIL011W 1284 uORF(11) -1220

uORF(29) -1185

uORF(17) -1115

uORF(2) -971

uORF(37) -1047

uORF(13) -900

uORF(11) -810

**uORF(7) -610**

uORF(55) -672

uORF(130) -716

**uORF(3) -316**

uORF(23) -265

uORF(75) -228

YIL118W 1875 uORF(15) -1854

uORF(7) -1788

uORF(39) -1775

**uORF(3) -1617**

uORF(20) -1542

uORF(17) -1508

**uORF(3) -1416**

uORF(31) -1423

uORF(52) -1284

uORF(26) -1195

uORF(35) -1148

uORF(12) -1069

**uORF(3) -978**

uORF(1) -925

**uORF(8) -863**

uORF(36) -886

uORF(31) -840

uORF(22) -776

uORF(10) -727

**uORF(4) -644**

uORF(18) -669

uORF(1) -602

uORF(26) -637

uORF(40) -507

**uORF(6) -347**

uORF(19) -237

**uORF(9) -151**

YIL119C 1875 uORF(6) -1838

uORF(11) -1848

uORF(19) -1790

uORF(59) -1809

**uORF(6) -1630**

uORF(16) -1590

uORF(76) -1727

uORF(8) -1316

**uORF(4) -1188**

uORF(33) -1234

uORF(12) -1167

uORF(33) -1070

uORF(16) -953

uORF(3) -900

uORF(31) -802

uORF(30) -749

uORF(9) -609

uORF(12) -530

uORF(13) -460

**uORF(4) -310**

**uORF(4) -269**

uORF(10) -103

YIL166C 1152 uORF(16) -1122

**uORF(4) -1065**

uORF(8) -1072

uORF(26) -1118

**uORF(9) -978**

uORF(2) -757

uORF(8) -739

uORF(3) -692

uORF(25) -723

uORF(15) -470

**uORF(4) -323**

uORF(25) -243

**uORF(6) -139**

uORF(45) -197

YJL036W 1489 uORF(13) -1485

**uORF(5) -1343**

uORF(6) -1226

uORF(38) -1099

uORF(8) -958

uORF(14) -948

**uORF(7) -783**

uORF(49) -667

**uORF(4) -445**

**uORF(3) -302**

uORF(25) -232

**uORF(8) -162**

YJL105W 1728 uORF(12) -1662

uORF(5) -1637

uORF(4) -1589

uORF(29) -1529

uORF(21) -1393

uORF(13) -1328

uORF(4) -1297

uORF(34) -1269

uORF(13) -1129

uORF(24) -1073

uORF(4) -999

uORF(43) -1027

**uORF(5) -872**

**uORF(7) -842**

uORF(13) -825

**uORF(6) -760**

**uORF(6) -703**

uORF(26) -743

**uORF(9) -628**

uORF(1) -584

uORF(48) -624

uORF(1) -408

**uORF(8) -400**

uORF(66) -344

YJL160C 1624 uORF(8) -1587

**uORF(5) -1395**

**uORF(3) -1332**

uORF(13) -1280

uORF(23) -1224

uORF(9) -1171

uORF(6) -1121

uORF(22) -1065

uORF(2) -984

uORF(38) -964

uORF(20) -848

uORF(15) -780

uORF(15) -761

YJL161W 1616 uORF(32) -1532

uORF(3) -1429

uORF(1) -1262

uORF(15) -1067

uORF(52) -1165

uORF(3) -946

uORF(17) -930

uORF(40) -883

uORF(2) -760

uORF(17) -737

uORF(26) -649

uORF(23) -567

uORF(25) -515

uORF(17) -405

uORF(13) -379

**uORF(6) -312**

uORF(42) -247

**uORF(7) -106**

uORF(4) -47

YJL200C 2030 uORF(4) -1974

uORF(26) -1941

uORF(32) -1718

uORF(33) -1687

uORF(21) -1589

uORF(1) -1442

uORF(18) -1482

uORF(7) -1425

uORF(17) -1352

uORF(12) -1236

uORF(108) -1369

uORF(6) -1013

uORF(18) -807

uORF(54) -908

uORF(1) -711

uORF(19) -751

uORF(17) -731

**uORF(5) -661**

uORF(26) -665

**uORF(5) -588**

**uORF(5) -542**

uORF(32) -486

uORF(14) -323

uORF(27) -354

**uORF(8) -137**

uORF(13) -147

YKL067W 1734 uORF(3) -1686

uORF(9) -1600

uORF(3) -1570

uORF(12) -1583

uORF(16) -1548

uORF(14) -1501

uORF(8) -1478

uORF(7) -1438

uORF(23) -1369

uORF(39) -1413

uORF(23) -1269

uORF(32) -1288

uORF(28) -1075

uORF(1) -943

uORF(17) -983

uORF(15) -963

uORF(14) -919

**uORF(4) -864**

uORF(23) -835

uORF(15) -733

uORF(17) -726

**uORF(4) -641**

uORF(15) -657

**uORF(8) -534**

uORF(1) -481

uORF(5) -467

uORF(3) -398

uORF(40) -463

uORF(49) -329

**uORF(7) -183**

YKL096W 1601 uORF(11) -1573

uORF(3) -1427

uORF(2) -1376

uORF(20) -1404

uORF(7) -1324

uORF(23) -1331

uORF(19) -1293

uORF(21) -978

uORF(36) -1012

uORF(16) -923

uORF(3) -852

uORF(2) -788

uORF(39) -862

uORF(21) -804

uORF(17) -708

**uORF(5) -612**

uORF(18) -641

uORF(24) -616

uORF(19) -582

**uORF(7) -411**

uORF(1) -198

YKL109W 2347 uORF(38) -2221

uORF(21) -2145

uORF(2) -2083

uORF(18) -2117

uORF(12) -2043

uORF(22) -1972

uORF(18) -1907

uORF(23) -1851

uORF(5) -1752

uORF(25) -1618

uORF(84) -1706

uORF(21) -1513

uORF(36) -1385

uORF(2) -1278

uORF(2) -1216

uORF(9) -1129

uORF(8) -1086

uORF(28) -1136

uORF(5) -1027

uORF(50) -1031

uORF(29) -811

uORF(14) -674

uORF(20) -541

uORF(47) -569

uORF(22) -472

uORF(11) -423

**uORF(9) -250**

**uORF(3) -62**

YKL182W 1030 uORF(2) -930

uORF(26) -974

uORF(42) -1018

uORF(1) -868

uORF(68) -888

uORF(69) -830

**uORF(6) -142**

YLR399C 1022 uORF(15) -1016

**uORF(7) -956**

uORF(40) -969

**uORF(7) -866**

**uORF(8) -761**

uORF(14) -731

uORF(70) -828

uORF(18) -619

**uORF(8) -263**

uORF(40) -259

YLR401C 1119 uORF(18) -1072

uORF(12) -967

**uORF(8) -917**

**uORF(4) -860**

uORF(38) -921

uORF(1) -771

uORF(10) -716

uORF(10) -644

uORF(11) -622

**uORF(5) -585**

uORF(17) -547

**uORF(7) -491**

uORF(10) -446

uORF(1) -394

uORF(15) -257

**uORF(9) -210**

uORF(39) -223

uORF(2) -71

**uORF(3) -56**

uORF(22) -99

YML043C 1021 uORF(5) -909

uORF(11) -879

uORF(39) -917

uORF(39) -895

uORF(39) -776

uORF(14) -655

**uORF(4) -549**

uORF(11) -494

uORF(22) -480

uORF(17) -433

uORF(2) -368

uORF(36) -277

uORF(2) -170

uORF(55) -249

uORF(4) -15

YMR016C 1896 uORF(10) -1883

uORF(1) -1715

uORF(22) -1753

uORF(33) -1746

**uORF(4) -1643**

uORF(3) -1386

uORF(9) -1378

uORF(14) -1359

uORF(16) -1230

uORF(56) -1201

uORF(1) -963

uORF(30) -851

uORF(3) -747

uORF(71) -931

uORF(1) -657

uORF(36) -606

uORF(12) -399

**uORF(8) -360**

uORF(21) -325

YMR145C 1006 uORF(22) -995

uORF(12) -930

**uORF(6) -895**

uORF(28) -748

uORF(28) -678

uORF(44) -662

uORF(19) -471

**uORF(4) -408**

uORF(74) -497

uORF(71) -418

**uORF(4) -165**

YMR280C 1010 **uORF(5) -922**

**uORF(4) -877**

uORF(2) -832

uORF(1) -718

uORF(25) -642

uORF(12) -589

**uORF(8) -551**

uORF(13) -486

uORF(17) -454

**uORF(5) -240**

**uORF(4) -201**

uORF(27) -244

YNL068C 1128 uORF(15) -1094

uORF(33) -1125

uORF(14) -1036

uORF(14) -886

uORF(28) -778

**uORF(8) -651**

uORF(20) -596

**uORF(4) -524**

uORF(42) -495

**uORF(7) -294**

uORF(68) -431

uORF(3) -12

YNR016C 1539 uORF(6) -1447

uORF(2) -1379

uORF(7) -1340

uORF(55) -1417

**uORF(3) -1105**

uORF(91) -1253

uORF(24) -997

uORF(2) -813

uORF(53) -788

uORF(39) -714

**uORF(3) -342**

YNR017W 1539 uORF(13) -1364

uORF(15) -1266

uORF(5) -1222

**uORF(7) -1144**

uORF(37) -1200

**uORF(3) -884**

uORF(1) -828

uORF(23) -761

uORF(19) -680

uORF(35) -624

uORF(25) -545

uORF(32) -487

uORF(25) -420

YNR055C 1431 uORF(9) -1430

uORF(11) -1212

uORF(17) -1216

uORF(18) -1205

uORF(12) -1023

uORF(12) -929

**uORF(7) -894**

uORF(11) -890

uORF(11) -865

**uORF(9) -660**

**uORF(7) -647**

uORF(31) -703

uORF(14) -620

**uORF(7) -579**

**uORF(7) -505**

**uORF(6) -216**

YOL100W 1317 uORF(2) -1281

uORF(46) -1304

uORF(32) -1107

uORF(15) -956

uORF(99) -1189

uORF(21) -948

**uORF(7) -861**

uORF(2) -800

**uORF(8) -628**

uORF(11) -599

uORF(26) -589

**uORF(4) -512**

uORF(14) -519

uORF(12) -505

**uORF(9) -338**

**uORF(7) -81**

YOL119C 1190 uORF(7) -944

uORF(13) -952

uORF(30) -972

uORF(18) -820

uORF(3) -724

uORF(3) -668

uORF(15) -664

**uORF(7) -623**

uORF(15) -610

**uORF(6) -566**

uORF(12) -446

uORF(102) -556

uORF(45) -308

**uORF(4) -181**

**uORF(8) -158**

YOL130W 1043 uORF(5) -957

uORF(13) -952

uORF(5) -899

uORF(22) -845

**uORF(8) -755**

**uORF(9) -742**

uORF(16) -728

**uORF(9) -648**

**uORF(3) -611**

**uORF(6) -482**

uORF(27) -453

uORF(40) -472

**uORF(8) -328**

uORF(20) -309

**uORF(5) -103**

YPL034W 1026 **uORF(8) -892**

**uORF(5) -855**

uORF(40) -711

uORF(13) -592

uORF(20) -526

uORF(12) -470

uORF(11) -425

uORF(11) -381

uORF(49) -448

uORF(2) -297

uORF(20) -302

uORF(36) -210

**uORF(4) -92**

**uORF(3) -78**

**uORF(8) -57**

uORF(9) -38

YPR091C 1090 uORF(16) -1088

uORF(18) -1041

uORF(16) -816

**uORF(5) -769**

**uORF(7) -711**

uORF(32) -674

uORF(14) -579

uORF(3) -466

**uORF(8) -368**

uORF(58) -480

**uORF(4) -274**

uORF(28) -335

**uORF(5) -244**

uORF(1) -198

uORF(26) -132

YPR149W 1778 uORF(38) -1761

uORF(19) -1682

uORF(16) -1594

uORF(19) -1500

uORF(37) -1523

uORF(19) -1391

uORF(37) -1431

uORF(2) -1321

**uORF(4) -1179**

uORF(41) -1196

uORF(75) -1064

**uORF(7) -818**

uORF(24) -859

uORF(13) -776

uORF(38) -828

**uORF(6) -725**

**uORF(6) -712**

uORF(26) -689

uORF(45) -685

**uORF(4) -481**

**uORF(6) -440**

uORF(22) -344
