# Supplementary material for: Identification of putative regulatory upstream ORFs in the yeast genome using heuristics and evolutionary conservation
Source: BMC Bioinformatics. 2007 Aug 8;8:295. doi: 10.1186/1471-2105-8-295 (PMC1964767; doi:10.1186/1471-2105-8-295)
Supplement: Additional file 3 — uORFs identified in verified 5'-UTRs by Zhang and Dietrich [19]. Numbering of uORFs is 5' to 3'. [file 1471-2105-8-295-S3.doc]

**Additional file 3.**

| Gene | Conserved uORFs/total number of uORFs; number of species with uORF conservation/number of species with orthologous gene | Comment |
| --- | --- | --- |
| *IMD1* | NA | no gene orthologues |
| RDH54 | NA/5; NA | not conserved |
| *AMN1* | 1/1;0/5 | too small but conserved, not the highest score |
| *YCL007C* | 0/4;0/3 | not conserved |
| *HNT1* | NA/4;NA | not conserved |
| *MAF1* | 0/1;0/6 | not conserved |
| *AGE1* | 2/6;2/6 | OK |
| *PIC2* | 1/1;7/7 | OK |
| *YER130C* | 0/1:0/6 | not conserved |
| *BUR6* | NA/2;NA | not conserved |
| *YGL059W* | NA/1;NA | not conserved |
| *PCL5* | 2/2;6/6 | OK |
| *LYS12* | 0/1;0/7 | not conserved |
| *MTR2* | 0/5;0/7 | not conserved |
| *MST1* | NA/1;NA | not conserved |
| *URA5* | 1/1;4/6 | -50 rule, not the highest score |
| *YMR009W* | 0/4;0/7 | not conserved |
| *YMR040W* | 0/3;0/5 | not conserved |
| *IPI3* | 0/1;0/4 | not conserved |
| *YNR034W-A* | NA/1;NA | ATG does not exist |
| *CRS5* | 0/1;0/5 | not conserved |
| *YOR051C* | 0/3;0/5 | not conserved |
| *ODC2c* | 0/8;0/5 | not conserved |
| *RHO1* | NA/1;NA | not conserved |
