# Supplementary material for: Identification of putative regulatory upstream ORFs in the yeast genome using heuristics and evolutionary conservation
Source: BMC Bioinformatics. 2007 Aug 8;8:295. doi: 10.1186/1471-2105-8-295 (PMC1964767; doi:10.1186/1471-2105-8-295)
Supplement: Additional file 4 — uORFs predicted to be functional by Zhang and Dietrich [17]. Numbering of uORFs 5' to 3'. [file 1471-2105-8-295-S4.doc]

**Additional file 4.**

| Gene | Conserved uORFs/total number of uORFs; number of species with uORF conservation/number of species with orthologous gene | Comment |
| --- | --- | --- |
| *APC2* | 0/1; 0/5 | Low cf value, –50 rule |
| ARV1 | 0/3; 0/4 | uORF2 has intermediate score (0.5), other have negative score |
| *AVT2* | 0/1; 0/3 | uORF has a low cf because of –50 rule |
| *ECM7* | 0/1; 0/6 | uORF in –50 region (within a longer uORF). Gene is in the long top list (252 genes), because of some other uORF with good score and conservation |
| *FOL1* | 1/1; 4/6 | OK |
| *HEM3* | 1/1; 6/6 | OK |
| *IMD4* | 0/1; 0/4 | Low score, not conserved |
| *MBR1* | 1/1; 3/3 | OK |
| *MKK1* | 1/1; 4/6 | OK |
| *RPC11* | 1/1; 5/6 | OK |
| *SLM2* | 2/3; 4/4 | 2/3 conserved; uORF2, uORF3 have a high score and are conserved |
| *SPE4* | 0/1; 0/7 | Low cf, –50 rule |
| *SPH1* | 1/1; 0/4 | Low cf –50 rule |
| *TPK1* | 0/1; 0/4 | uORF has negative cf, because of –50 rule |
| *WSC3* | 1/1; 3/3 | OK |
