# Supplementary material for: Identification of putative regulatory upstream ORFs in the yeast genome using heuristics and evolutionary conservation
Source: BMC Bioinformatics. 2007 Aug 8;8:295. doi: 10.1186/1471-2105-8-295 (PMC1964767; doi:10.1186/1471-2105-8-295)
Supplement: Additional file 5 — Attribute values of the expert system and their certainty factor [file 1471-2105-8-295-S5.doc]

**Additional file 5**.

| Length of uORF (L) | Number of uORFs (N) | Position of uORF (D) | Certainty factor (cf) |
| --- | --- | --- | --- |
| L<3 |  |  | -0.5 |
| 4<=L<=6 |  |  | 0.8 |
| 7<=L<=10 |  |  | 0.5 |
| 11<=L<=15 |  |  | 0.1 |
| L>=16 |  |  | -0.6 |
| 4<=L<=10 | 1<=N<=10 |  | 0.8 |
| 11<=L<=16 | 11<=N<=20 |  | 0.1 |
| L>=17 | N>=21 |  | -0.6 |
|  |  | 0<=D<=50 | -0.9 |
| 4<=L<=10 |  | 51<=D<=250 | 0.9 |
| L>=11 |  | 51<=D<=250 | -0.2 |
|  |  | D>=251 | 0.1 |
